# Kupffer Cells Hasten Resolution of Liver Immunopathology in Mouse Models of Viral Hepatitis

**DOI:** 10.1371/journal.ppat.1002061

**Published:** 2011-06-02

**Authors:** Giovanni Sitia, Matteo Iannacone, Roberto Aiolfi, Masanori Isogawa, Nico van Rooijen, Cristina Scozzesi, Marco E. Bianchi, Ulrich H. von Andrian, Francis V. Chisari, Luca G. Guidotti

**Affiliations:** 1 Division of Immunology, Transplantation and Infectious Diseases, San Raffaele Scientific Institute, Milan, Italy; 2 Immune Disease Institute and Department of Pathology, Harvard Medical School, Boston, Massachusetts, United States of America; 3 Department of Immunology & Microbial Sciences, The Scripps Research Institute, La Jolla, California, United States of America; 4 Department of Molecular Cell Biology, Free University Medical Center, Amsterdam, The Netherlands; 5 DiaPro Diagnostic Bioprobes, Milan, Italy; 6 Vita-Salute San Raffaele University, Milan, Italy; 7 Division of Genetics and Cell Biology, San Raffaele Scientific Institute, Milan, Italy; University of Southern California, United States of America

## Abstract

Kupffer cells (KCs) are widely considered important contributors to liver injury during viral hepatitis due to their pro-inflammatory activity. Herein we utilized hepatitis B virus (HBV)-replication competent transgenic mice and wild-type mice infected with a hepatotropic adenovirus to demonstrate that KCs do not directly induce hepatocellular injury nor do they affect the pathogenic potential of virus-specific CD8 T cells. Instead, KCs limit the severity of liver immunopathology. Mechanistically, our results are most compatible with the hypothesis that KCs contain liver immunopathology by removing apoptotic hepatocytes in a manner largely dependent on scavenger receptors. Apoptotic hepatocytes not readily removed by KCs become secondarily necrotic and release high-mobility group box 1 (HMGB-1) protein, promoting organ infiltration by inflammatory cells, particularly neutrophils. Overall, these results indicate that KCs resolve rather than worsen liver immunopathology.

## Author Summary

Kupffer cells (KCs), the resident macrophages of the liver, are considered important contributors to liver injury during viral hepatitis due to their pro-inflammatory activity. Herein we utilized two different mouse models of viral hepatitis (where liver damage is triggered, as during viral hepatitis in humans, by virus-specific CD8 T cells) to show that KCs do not directly induce liver injury nor do they affect the pathogenic potential of virus-specific CD8 T cells. Instead, KCs limit the severity of liver immunopathology. Mechanistically, our results are most compatible with the hypothesis that KCs contain liver immunopathology by removing dying hepatocytes. Dying hepatocytes not readily removed by KCs release high-mobility group box 1 (HMGB-1) protein, promoting organ infiltration by inflammatory cells, particularly neutrophils. These results indicate that KCs resolve rather than worsen liver disease.

## Introduction

Kupffer cells (KCs) are non-parenchymal cells that account for approximately 15% of the total liver cell population and constitute 80%–90% of the tissue-resident macrophages in the whole body [1]. Due to their intravascular (sinusoidal) localization, KCs have long been studied as scavenger cells that physiologically remove particulate material (e.g. aged blood cells, immune complexes and gut-derived bacterial products) from the portal circulation [1]. In recent years KCs have been implicated in the pathogenesis of an assortment of inflammatory liver diseases, including viral hepatitis [2], . Accordingly, the current dogma regarding the role of KCs in hepatitis B virus (HBV) or hepatitis C virus (HCV) pathogenesis considers these cells as important contributors to liver injury [4], [5]. As the host tropism of HBV and HCV is limited to humans and human primates [6], this dogma has been largely inferred from mouse studies in which KCs activated by a variety of stimuli (including the engulfment of apoptotic hepatocytes) were shown to i) promote the intrahepatic accumulation of pathogenic T cells and/or ii) express/produce inflammatory mediators that are directly toxic for the hepatocyte (e.g. tumor necrosis factor (TNF)-α, FasL, reactive oxygen species, etc) [2], [3], [7]. It is worth noting, however, that most of these studies were performed either by infecting mice with pathogens that preferentially replicate inside KCs (e.g. cytomegalovirus [8], [9], influenza [10], lymphocytic choriomeningitis virus [11], listeria [12] or leishmania [13]) or by injecting mice with lymphocyte mitogens (such as Staphylococcus enterotoxin B and Concanavalin A [14], [15]) that cause massive intrahepatic expansion of activated CD4 T cells. Since HBV and HCV infect almost exclusively the hepatocyte and since liver damage during these infections is primarily a consequence of the virus-specific CD8 T cell response [6], we decided to assess the role of KCs in more relevant animal models.

In one model effector HBV-specific CD8 T cells are adoptively transferred into immunocompetent transgenic mice that replicate HBV at high levels in the hepatocyte [16]–[21]. In a second model wild-type mice previously immunized with a plasmid encoding β-galactosidase (β-Gal) are infected with a β-Gal-expressing hepatocyte-tropic adenovirus [21], [22]. Both models revealed that KCs have no impact on the ability of virus-specific CD8 T cells to home to the liver, recognize antigen and kill hepatocytes, nor do they significantly act as effector cells to destroy the hepatocytes. Instead, KCs reduce the overall severity of T cell-mediated immunopathology by removing apoptotic hepatocytes from the liver.

## Results

### Treatment with clodronate liposomes (Clo-L) reduces KC number and liver phagocytic function

HBV replication-competent transgenic mice or C57BL/6 mice were injected intravenously with Clo-L. This treatment effectively eliminated F4/80^+^ KCs within 2 days and for at least 1 week after injection (Figure S1 and [23], [24]), without reducing the number of liver CD11c^high^ dendritic cells (DCs, Figure S1) or circulating Gr-1^high^ CD11b^+^ polymorphonuclear neutrophils (PMNs) and Ly-6C^+^ monocytes (Figure S1). Intravenously injected fluorescent beads failed to accumulate in the liver of Clo-L-treated mice (not shown), while they were readily up-taken by KCs from control animals (Figure S1). As the former mice also showed delayed clearance of beads from the circulation (Figure S1), these results indicate that Clo-L significantly reduced liver phagocytic function.

### KC depletion exacerbates liver immunopathology in models of acute viral hepatitis

**Figure 1:**
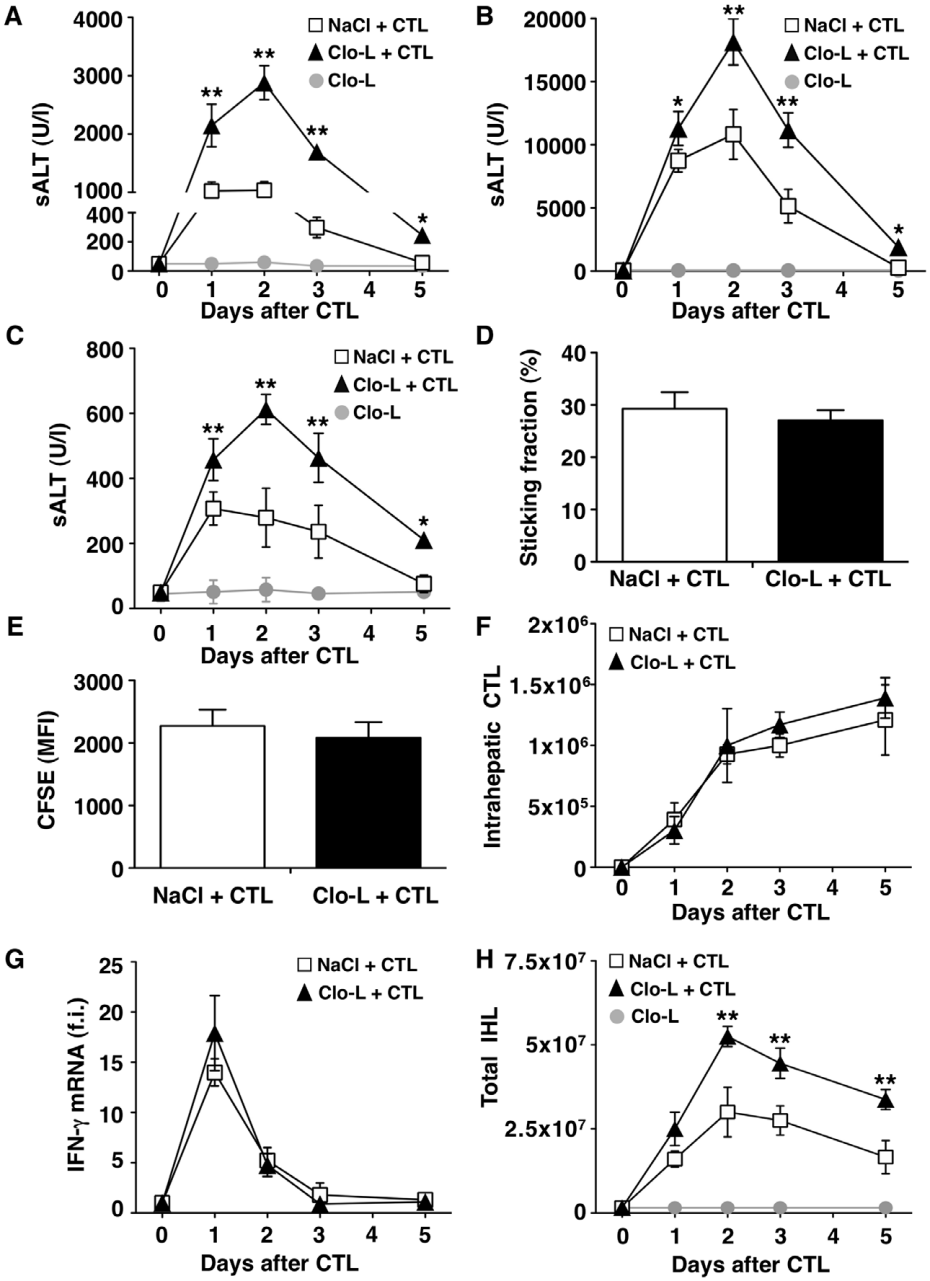
KCs limit liver immunopathology in HBV replication-competent transgenic mice, without affecting the number or function of virus-specific effector CD8 T cells (CTL). (A, B and C) Mean sALT activity (units/liter) measured at the indicated time points after intravenous injection of 10^7^ (A), 5×10^7^ (B) or 0.5×10^7^ (C) HBV-specific effector CD8 T cells (CTL) in HBV replication-competent transgenic mice that received the indicated treatment. *n* = 6. (D) Sticking fraction (the percentage of cells that arrested for ≥30 s in the total flux) of HBV-specific CTL in liver sinusoids of control (white) or Clo-L-treated (black) HBV replication-competent transgenic mice. *n* = 3. (E) Mean fluorescent intensity of CFSE-labeled HBV-specific CTL recovered from control (white) or Clo-L-treated (black) HBV replication-competent transgenic livers, two days after intravenous injection of 10^7^ HBV-specific CTL. *n* = 6. (F) Absolute number of HBV-specific CTL recovered from control (white) or Clo-L-treated (black) HBV replication-competent transgenic livers, at the indicated time points after intravenous injection of 10^7^ HBV-specific CTL. *n* = 6. (G) IFN-γ mRNA expression in control (white) or Clo-L-treated (black) HBV replication-competent transgenic livers, at the indicated time points after intravenous injection of 10^7^ HBV-specific CTL. Results are expressed as fold induction (f.i.) over controls (i.e. mice injected with either NaCl or Clo-L alone), after normalization for the housekeeping gene L32. *n* = 6. (H) Absolute number of intrahepatic leukocytes (IHL) recovered from control (white) or Clo-L-treated (black) HBV replication-competent transgenic livers, at the indicated time points after intravenous injection of 10^7^ HBV-specific CTL. *n* = 6. All data are expressed as mean ± standard deviation and are representative of at least 3 independent experiments that gave similar results; differences between CTL-injected mice treated or not with Clo-L were not statistically significant unless otherwise indicated, * *p*<0.05, ** *p*<0.001.

HBV replication-competent transgenic mice were treated with either Clo-L or saline (NaCl) 3 days prior to the transfer of effector HBV-specific CD8 T cells. Additional groups of control mice were injected with NaCl, liposomes containing saline (NaCl-L) or Clo-L alone. As previously reported [17], injection of effector HBV-specific CD8 T cells (derived from immunized syngeneic non transgenic mice, see Methods) into saline-treated control mice caused a transient liver disease (monitored biochemically by measuring the serum activity of alanine aminotransferase [sALT], an enzyme that is released into the circulation by necrotic hepatocytes) that almost completely resolved 5 days after transfer (Figure 1A). No difference in disease severity (monitored by sALT activity [Figure S2] and liver histology [not shown]) was observed when NaCl-L was injected, instead of NaCl, prior to the transfer of effector HBV-specific CD8 T cells. As also previously reported [17], resolution of disease in this model is due to CD8 T cell-dependent down-regulation of viral antigens (see below and Discussion). Surprisingly Clo-L treatment, which by itself did not cause significant sALT elevation, markedly increased liver disease in CD8 T cell-injected mice at all time points (Figure 1A). This effect occurred independently of the number of effector HBV-specific CD8 T cells that were transferred (Figure 1, B and C). To exclude the possibility that Clo-L may prolong sALT half-life, we injected liver extracts with a known sALT content in either control or Clo-L-treated mice. sALT half-life in Clo-L-treated mice was not significantly different from that of controls (489 versus 538 minutes, respectively, Figure S3), indicating that sALT is a reliable marker of liver disease in Clo-L treated mice.

**Figure 2:**
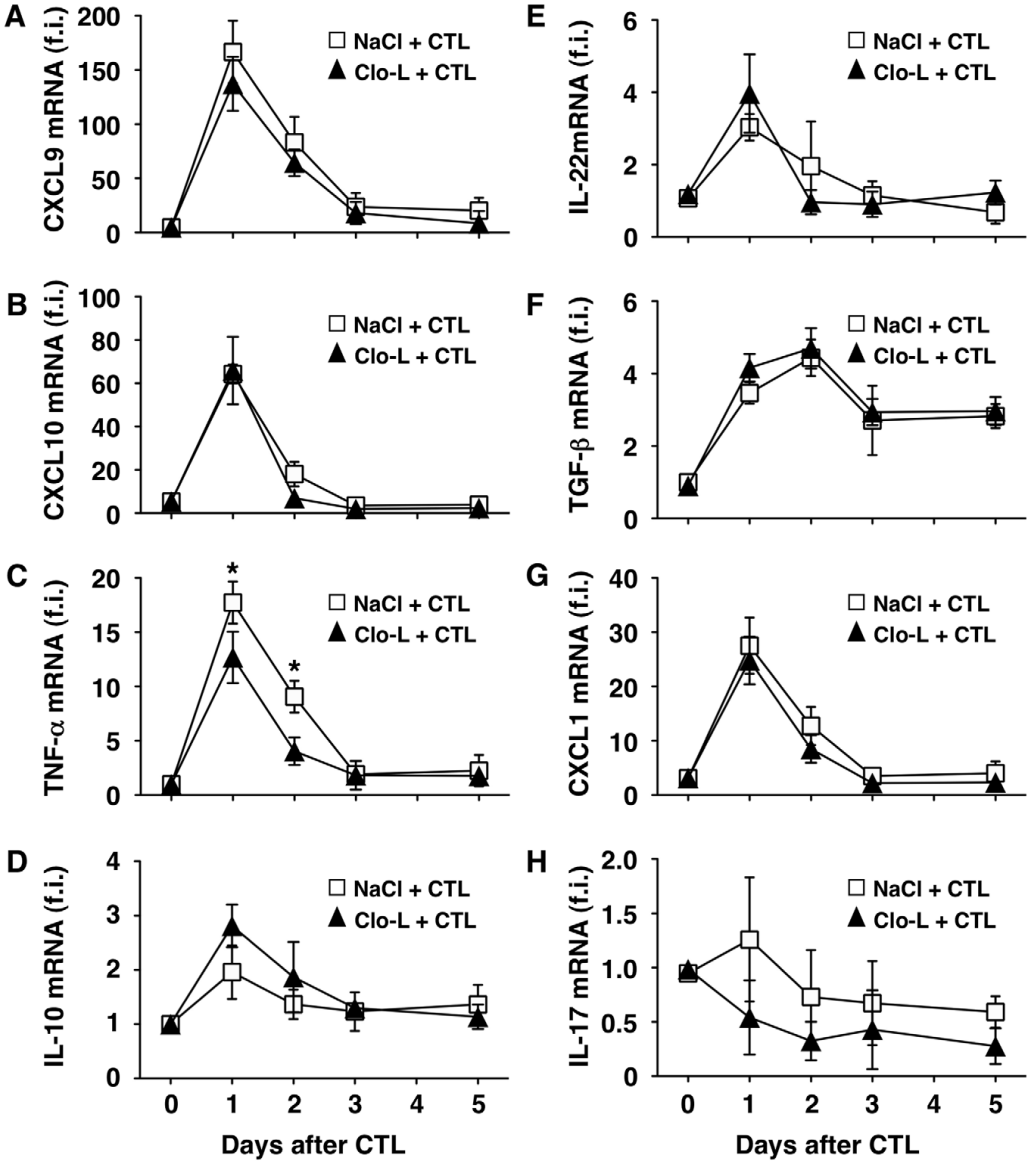
Clo-L treatment does not affect the hepatic gene expression of CXCL9, CXCL10, IL-10, IL-22, TGF-β, CXCL1 or IL-17 but reduces that of TNF-α. CXCL9 (A), CXCL10 (B), TNF-α (C), IL-10 (D), IL-22 (E), TGF-β (F), CXCL1 (G) or IL-17 (H) liver mRNA expression from mice described in the legend to Figure 1G. Results are expressed as fold induction (f.i.) over controls (i.e. mice injected with either NaCl or Clo-L alone), after normalization for the housekeeping gene L32. *n* = 6. Differences were not statistically significant unless otherwise indicated, * *p*<0.05.

As virus-specific CD8 T cells trigger liver disease in this model, we next asked whether KCs altered the pathogenic potential of these cells *in vivo*. To this end, we first performed intravital microscopic analysis to monitor the behavior of the transferred T cells. Approximately 30% of visualized intrahepatic HBV-specific CD8 T cells arrested and transiently contacted KCs (mean interaction time of 5±1 second) (Video S1). Adhesion of HBV-specific CD8 T cells to liver sinusoids did not require interaction with KCs as the percentage of CD8 T cells stably adhering (i.e. >30 s) to liver sinusoidal endothelial cells was similar between saline controls and Clo-L treated mice (Figure 1D, Video S2 and S3). The expansion and intrahepatic accumulation of the transferred T cells (Figure 1, E and F) and the relative increase (fold induction over mice injected with either NaCl or Clo-L alone) of liver mRNAs encoding for IFN-γ (a well-established marker of antigen recognition by HBV-specific CD8 T cells in this model [17], [25]) (Figure 1G) or CXCL9 and CXCL10 (two IFN-γ inducible chemokines most abundantly expressed by hepatocytes surrounding inflammatory foci [18]) (Figure 2, A and B) were also unaffected in Clo-L treated mice. The relative increase of liver mRNA encoding for TNF-α - another cytokine known to be produced by activated HBV-specific CD8 T cells in this model [17], [25] and that has been proposed to promote liver damage [26] - was reduced of about 33% and 55% at days 1 and 2, respectively, in CD8 T cell-injected mice treated with Clo-L (Figure 2C), indicating that KCs contributed to the production of this pro-inflammatory soluble mediator.

As mentioned earlier, liver disease in this model is transient because IFN-γ-dependent mechanisms rapidly eliminate viral gene products from the liver [17], [25], [27]. Accordingly, viral DNA, RNA and proteins disappeared from the liver of saline- and Clo-L-treated mice with comparable kinetics (not shown and Figure S4). Note that 5 days after CD8 T cell injection Clo-L-treated mice showed residual antigen reactivity in hepatocytes that appeared morphologically damaged (Figure S4), suggesting that these target cells were not readily removed from the liver. Importantly, the number of infiltrating intrahepatic leukocytes (IHL), the vast majority of which are antigen-non-specific inflammatory cells [18], was significantly increased in CD8 T cell-injected mice that were treated with Clo-L instead of NaCl (Figure 1H), while treatment with Clo-L alone increased neither sALT activity (Figure 1, A, B and C) nor IHL infiltration (Figure 1H). Together, the data indicate that Clo-L treatment exacerbates immunopathology independently of the number or function of intrahepatic HBV-specific CD8 T cells and is associated with a more abundant antigen-non-specific inflammatory cell infiltrate in the liver. Exacerbation of liver immunopathology in face of reduced numbers of KCs (and reduced levels of the TNF-α they produce also indicates that KCs play little or no role as effector cells in the destruction of hepatocytes in this system. Additionally, the intrahepatic expression levels of potentially anti-inflammatory/hepatoprotective cytokines such as IL-10, IL-22 or TGF-β [28]–[30] were not reduced in CD8 T cell-injected Clo-L-treated mice when compared to proper controls (Figure 2, D, E and F), indicating that the increased liver disease observed in the former animals was not associated to decreased production of cytoprotective factors.

**Figure 3:**
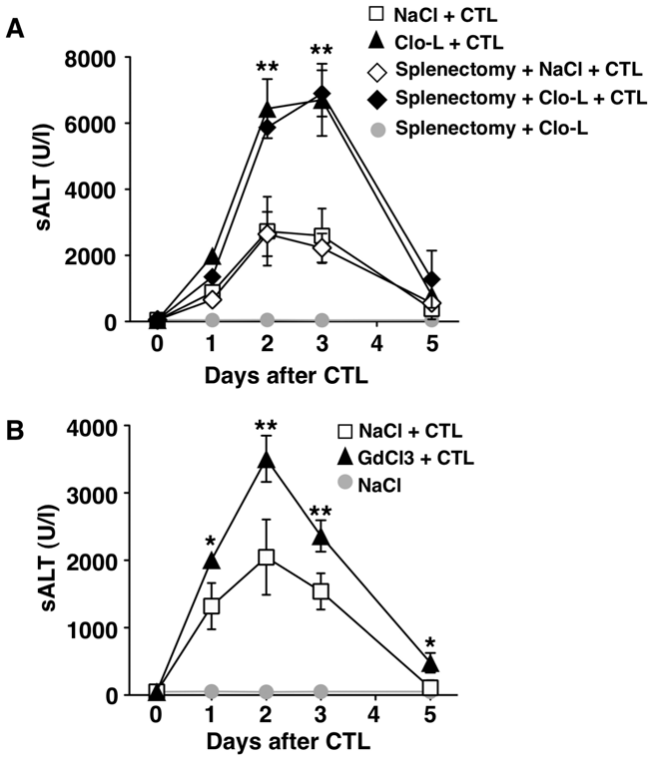
The effects of splenectomy and GdCl3 on liver disease severity. (A–B) Mean sALT activity (units/liter) measured at the indicated time points after intravenous injection of 10^7^ HBV-specific effector CD8 T cells (CTL) that received the indicated treatment. *n* = 6. All data are expressed as mean ± standard deviation and are representative of at least 2 independent experiments that gave similar results; note that no difference in sALT activity was detected between splenectomized and non-splenectomized animals at all time points after CTL injection; differences between CTL-injected mice treated or not with Clo-L or GdCl3 were not statistically significant unless otherwise indicated, ** *p*<0.001.

Notably, intravenous Clo-L treatment is not specific for KCs, but it apparently acts on other cell populations, particularly splenic mononuclear phagocytes [31], [32]. To rule out a contribution of splenocytes in our system, HBV replication-competent transgenic mice were splenectomized and treated with either Clo-L or saline prior to the transfer of effector HBV-specific CD8 T cells. Splenectomized animals showed a degree of liver disease severity that was virtually identical to that of non-splenectomized controls (Figure 3A), strongly suggesting that the capacity of Clo-L to exacerbate liver disease depends on depletion of KCs rather than depletion of splenic mononuclear phagocytes. Additional evidence supporting the notion that KC depletion is associated with liver disease exacerbation in this model comes from experiments where HBV replication-competent transgenic mice were administered with gadolinium chloride (GdCl3) prior to the transfer of CD8 T cells. GdCl3 is a rare earth metal that - like Clo-L - has been widely used to eliminate KCs in mice [33]–[35]. As shown in Figure S1 and Figure 3B, respectively, GdCl3 treatment reduced KC number by more than 75% (without reducing the frequency of circulating monocytes and PMNs, not shown) and caused a significant increase in sALT activity at all time points after CD8 T cell transfer.

**Figure 4:**
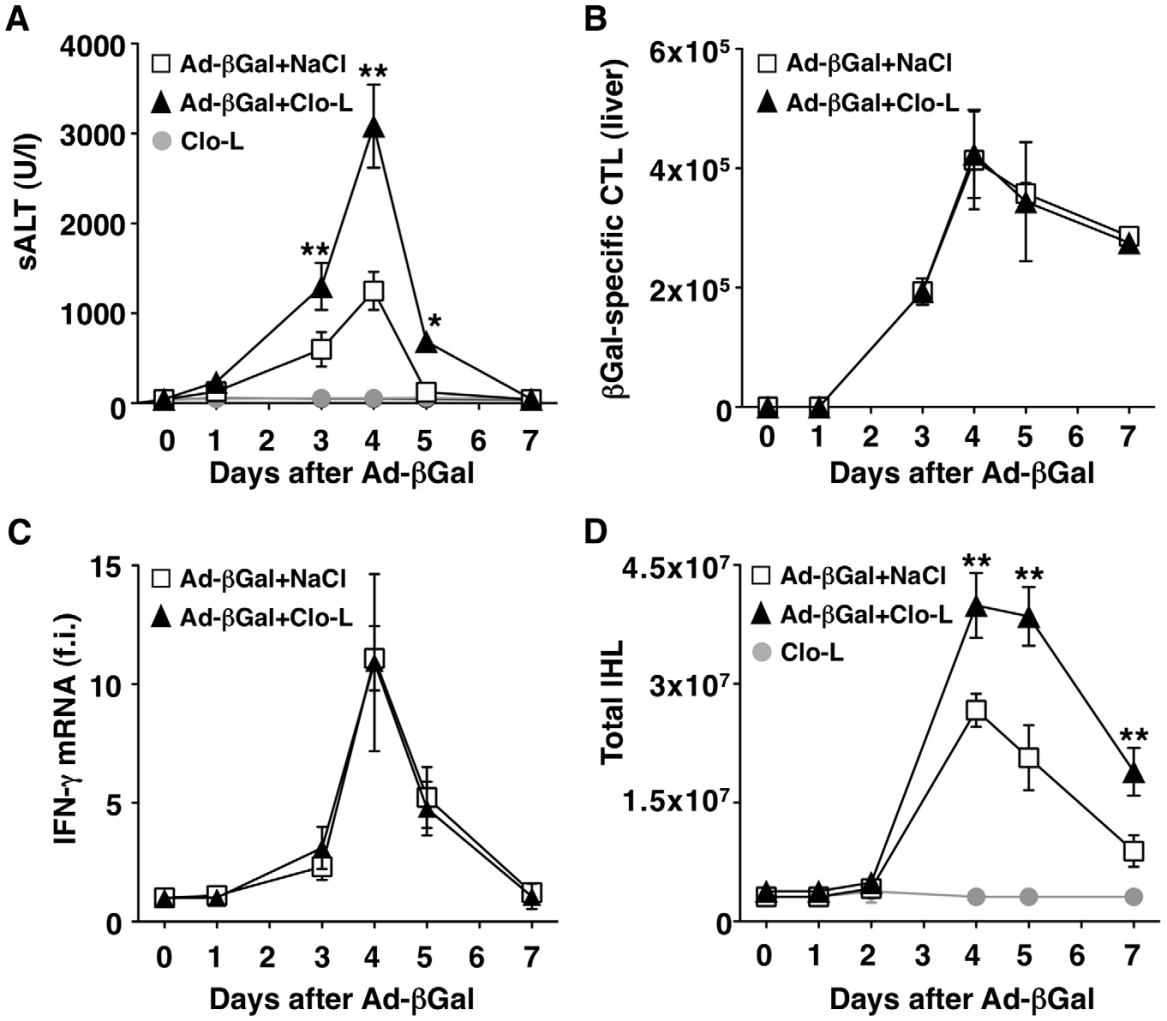
KCs limit liver immunopathology in adenovirus-infected mice, without affecting the number or function of virus-specific CTL. (A) Mean sALT activity (units/liter) measured at the indicated time points after infection with 10^9^ pfu of a β-Gal-expressing adenovirus (Ad-β-Gal) in mice that received the indicated treatment. *n* = 6. (B) Absolute number of β-Gal-specific CTL recovered from control (white) or Clo-L-treated (black) livers, at the indicated time points after infection with 10^9^ pfu of Ad-β-Gal. *n* = 6. (C) IFN-γ mRNA expression in control (white) or Clo-L-treated (black) livers, at the indicated time points after infection with 10^9^ pfu of Ad-β-Gal. Results are expressed as fold induction (f.i.) over controls (i.e. mice injected with either NaCl or Clo-L alone), after normalization for the house-keeping gene L32. *n* = 6. (D) Absolute number of intrahepatic leukocytes (IHL) recovered from control (white) or Clo-L-treated (black) livers, at the indicated time points after infection with 10^9^ pfu of Ad-β-Gal. *n* = 6. All data are expressed as mean ± standard deviation and are representative of at least 3 independent experiments that gave similar results; differences between Ad-β-Gal-infected mice treated or not with Clo-L were not statistically significant unless otherwise indicated, * *p*<0.05, ** *p*<0.001.

Next, we extended our study to a previously established infection model [21] in which liver immunopathology is triggered by endogenous β-Gal-specific CD8 T cells that recognize hepatocytes infected by a β-Gal-expressing, replication-deficient adenovirus (Ad-β-Gal). C57BL/6 mice were immunized intramuscularly with a β-Gal-expressing plasmid to generate β-Gal-specific CD8 T cells. Three weeks later the mice were grouped based on the frequency of circulating β-Gal-specific CD8 T cells and then infected with the hepatotropic Ad-β-Gal (10^9^ pfu/mouse) (see Methods). Clo-L treatment was carried out one day after Ad-β-Gal infection, when the percentage of β-Gal-expressing hepatocytes is maximal and viremia is no longer detectable ([21] and not shown). When compared to saline-treated controls, Clo-L-treated mice showed i) significantly higher sALT activity (Figure 4A), ii) comparable numbers of intrahepatic β-Gal-specific CD8 T cells (Figure 4B), iii) comparable amounts of liver IFN-γ mRNA (Figure 4C), and iv) increased organ infiltration of antigen non-specific leukocytes (Figure 4D). Thus, KC-related pathogenic events that are similar to those observed in HBV replication-competent transgenic mice (Figure 1) were also operative in this infection model of liver immunopathology.

### Impaired removal of apoptotic hepatocytes and focal hepatocellular necrosis in Clo-L- or GdCl3-treated mice

**Figure 5:**
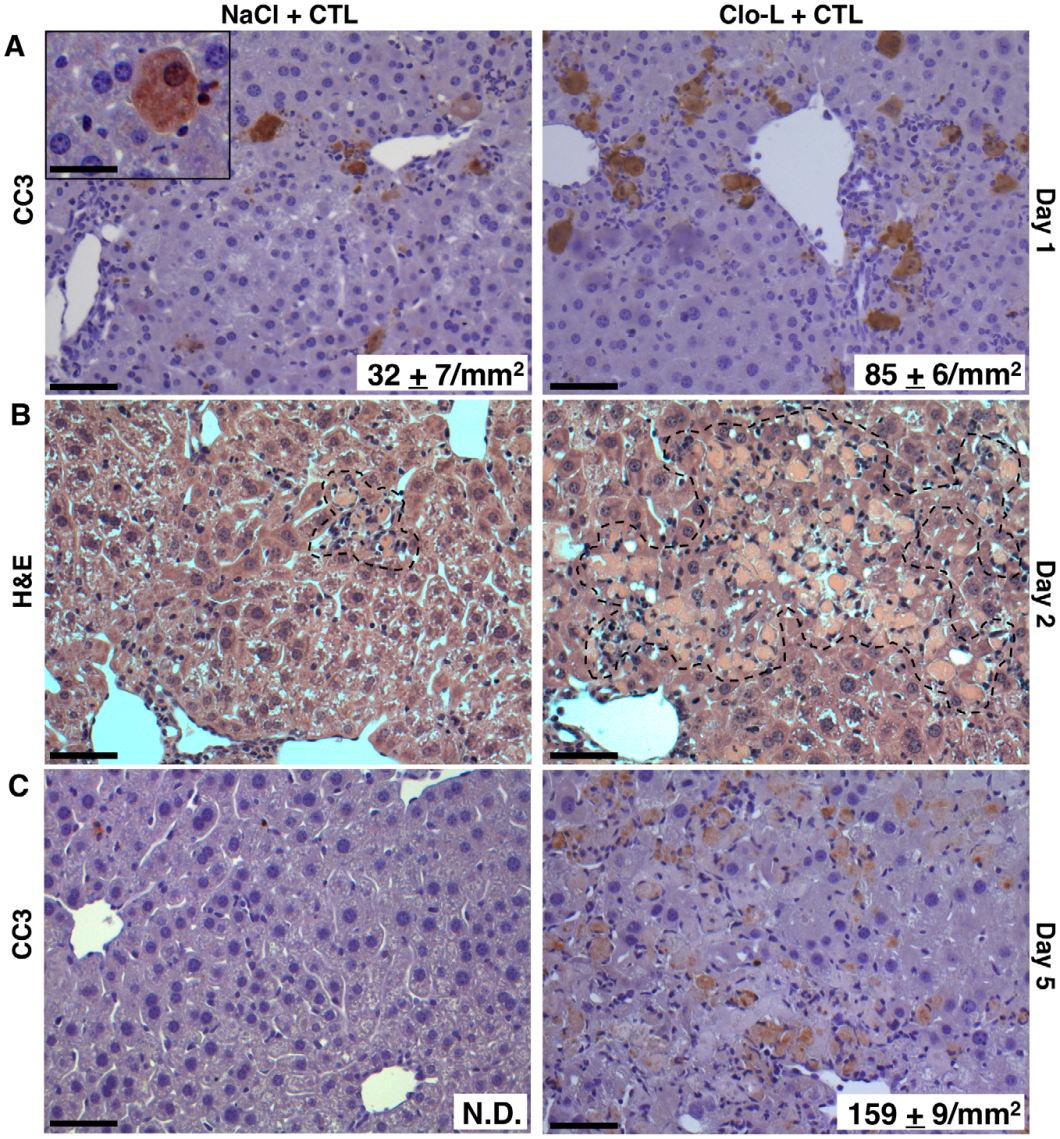
Impaired removal of apoptotic hepatocytes and focal hepatocellular necrosis in Clo-L-treated mice. (A,C) Representative immunohistochemical micrographs of control (left panels) or Clo-L-treated (right panels) HBV replication-competent transgenic livers, one (A) or five (C) days after intravenous injection of 10^7^ HBV-specific CTL. Cleaved caspase 3 (CC3) staining in brown. The number (mean ± standard deviation) of CC3^+^ hepatocytes was calculated in 100 high power fields (corresponding to about 4 mm^2^ of liver tissue) and is indicated in bottom right insets. N.D.: non detectable. Scale bars represent 150 µm or, in the case of the upper left inset, 20 µm. *n* = 6, *p*<0.05. (B) Representative micrographs of hematoxylin/eosin-stained control (left) or Clo-L-treated (right) HBV replication-competent transgenic livers, two days after intravenous injection of 10^7^ HBV-specific CTL. Broken line delineates necroinflammatory foci. Data are representative of at least 3 independent experiments that gave similar results.

We next set out to identify the mechanism by which KCs limit liver immunopathology. When we quantified morphometrically the number of hepatocytes that stained positive for cleaved caspase 3 (CC3, a marker of hepatocellular apoptosis) in either HBV replication-competent transgenic mice killed 1 day after CD8 T cell injection (Figure 5A) or C57BL/6 mice killed 3 days after Ad-β-Gal infection (not shown), we observed a ∼3-fold increase of CC3^+^ hepatocytes in Clo-L-treated mice compared with saline-treated controls, with similar results observed in GdCl3-treated animals (not shown). One day later, the number of apoptotic hepatocytes increased disproportionately in Clo-L- or GdCl3-treated mice, resulting in the formation of large necroinflammatory foci thzat also displayed evident focal hepatocellular necrosis and dropout (Figure 5B and not shown). By day 5 after CD8 T cell injection or Ad-β-Gal-infection, virtually no CC3^+^ hepatocytes were visible in saline-treated control animals, while these cells remained readily detectable in Clo-L- or GdCl3-treated mice (Figure 5C and not shown). These results indicate that the higher sALT levels observed in these latter animals (Figures 1, 3, 4 and S2) probably reflected the secondary necrosis of apoptotic hepatocytes that had not been removed due to the absence of KCs and, therefore, accumulated over time. That these higher sALT levels did not reflect the destruction of greater numbers of hepatocytes by the CD8 T cells was further suggested by the serum levels of albumin and bilirubin (two indicators of metabolic functions arising from healthy hepatocytes), which were virtually identical in the saline- and Clo-L-treated mice (Figure S5).

### HMGB-1 and the role of PMNs

**Figure 6:**
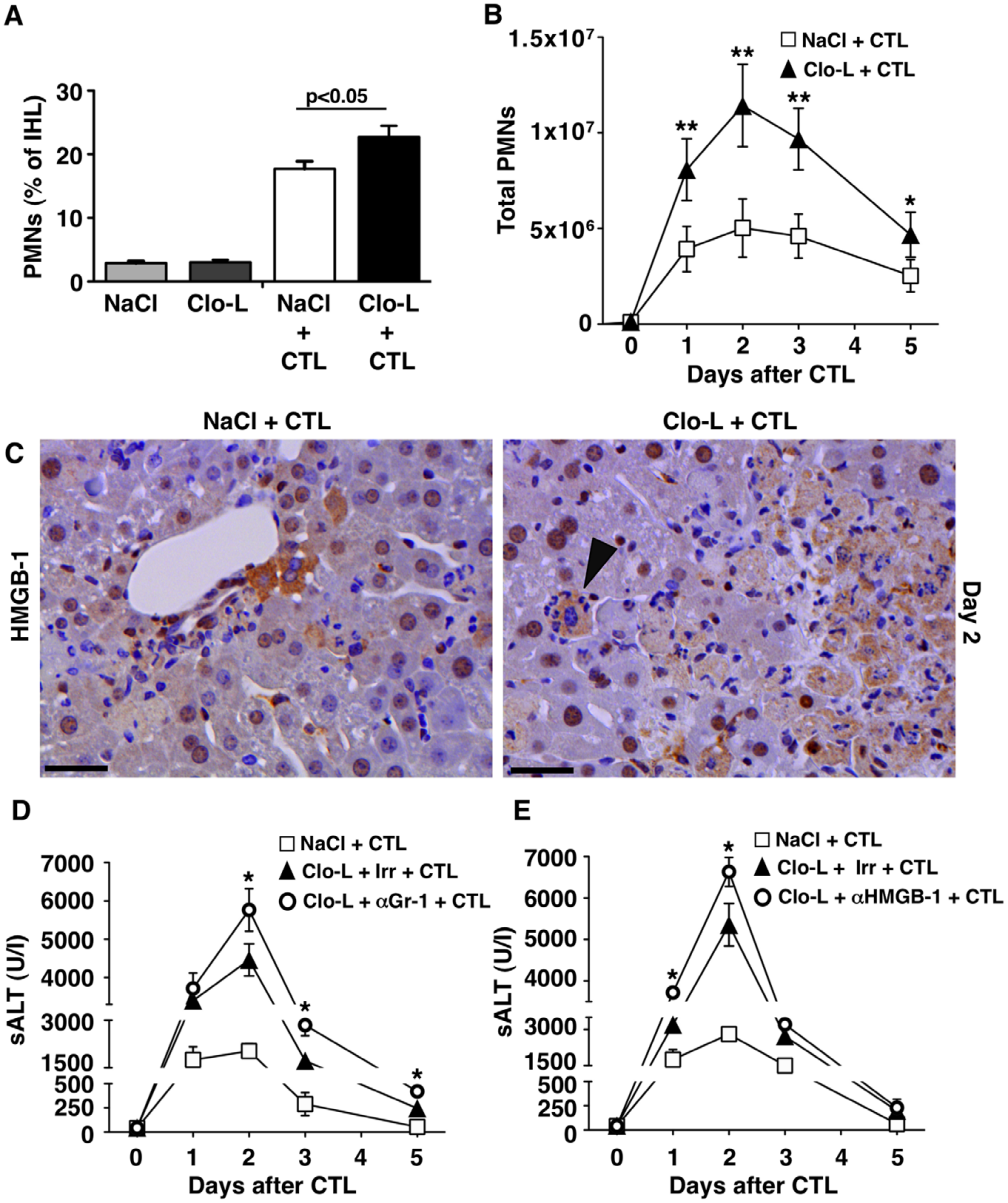
HMGB-1 and the role of PMNs. (A,B) Relative frequency (A) and absolute number (B) of Gr-1^high^ CD11b^+^ neutrophils (PMNs) recovered from the livers of mice that received the indicated treatment, 2 days (A) or at the indicated time point (B) after intravenous injection of 10^7^ HBV-specific CTL. (C) Representative immunohistochemical micrographs of control (left) or Clo-L-treated (right) HBV transgenic livers, two days after intravenous injection of 10^7^ HBV-specific CTL. HMGB-1 staining in brown. Note the nuclear-to-cytoplasm translocation of HMGB-1 in hepatocytes surrounded by PMNs (arrowhead). Scale bar represents 150 µm. *n* = 3 (D and E) Mean sALT activity (units/liter) measured at the indicated time points after intravenous injection of 10^7^ HBV-specific CTL in HBV transgenic mice that received the indicated treatment. Irr, irrelevant antibodies. *n* = 6. All data are expressed as mean ± standard deviation and are representative of at least 3 independent experiments that gave similar results; differences between CTL-injected mice treated or not with Clo-L (A,B) or between Clo-L- and CTL-injected mice treated or not with αGr-1 antibodies (E) were not statistically significant unless otherwise indicated, * *p*<0.05, ** *p*<0.001.*

**Figure 7:**
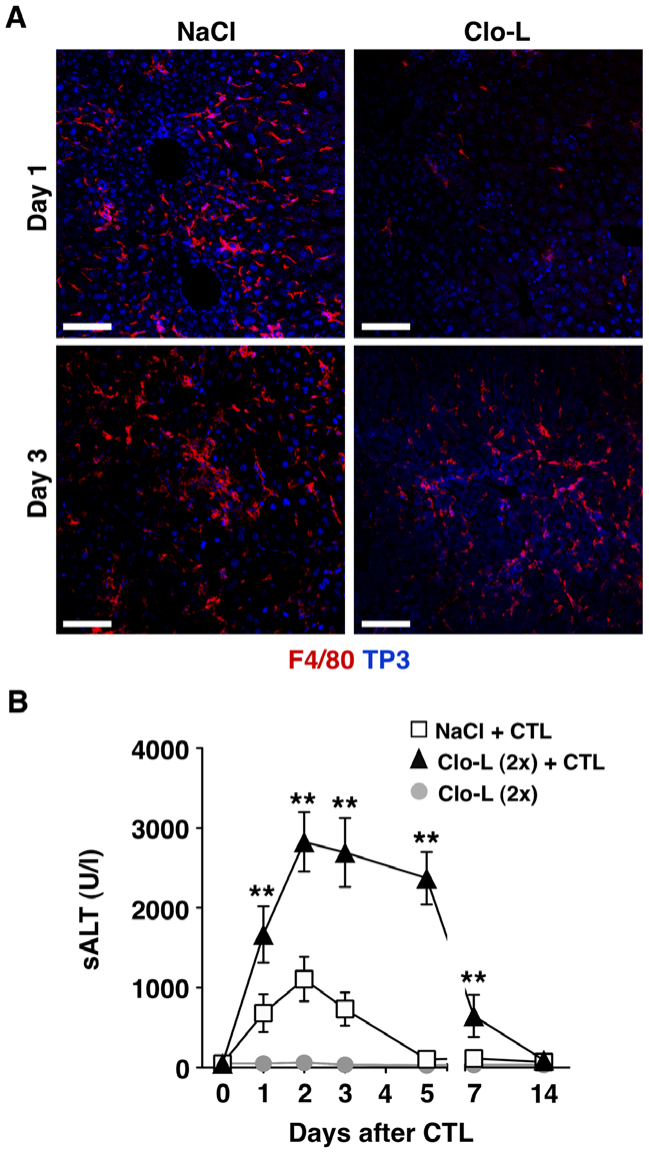
Recruitment of F4/80^+^ cells into the inflamed liver and prolongation of disease severity in CTL-injected mice receiving a second Clo-L injection. (A) Representative confocal micrographs of livers from NaCl- (left panels) or Clo-L-treated (right panels) HBV replication-competent transgenic mice one or three days after intravenous injection of 10^7^ HBV-specific CTL. Anti-F4/80 staining in red, TO-PRO-3 (TP3) staining of nuclei in blue. Scale bar represents 150 µm. *n* = 3. (B) Mean sALT activity (units/liter) measured at the indicated time points after intravenous injection of 10^7^ HBV-specific CTL in HBV replication-competent transgenic mice that received the indicated treatment. Clo-L was injected 3 days before and 3 days after CTL injection. *n* = 6. All data are expressed as mean ± standard deviation and are representative of at least 3 independent experiments that gave similar results; differences between CTL-injected mice treated (twice) or not with Clo-L were not statistically significant unless otherwise indicated, * *p*<0.05, ** *p*<0.001.

Cytometric analyses of the liver infiltrate in the mice described in Figures 1 and 4 revealed that the number of intrahepatic PMNs, a prominent intrahepatic inflammatory cell subset, was significantly higher in Clo-L-treated animals both in terms of relative frequency (Figure 6A and not shown) and, more importantly, absolute numbers (Figure 6B and not shown). The frequency/number of other abundant intrahepatic subsets such as CD8+ T cells (both antigen-specific and antigen-non-specific) and CD11c^high^ DCs (peak values of 9%/2.3×10^6^ and 4.7%/1.2×10^6^ respectively) did not change as a function of Clo-L treatment (not shown). Since HMGB-1 translocation in damaged hepatocytes has been previously linked to liver PMN recruitment in this model [36], we next monitored HMGB-1 expression in the above-mentioned livers. Large numbers of cytoplasmic HMGB-1^+^ hepatocytes juxtaposed to or surrounded by PMNs were found in the livers of Clo-L-treated HBV replication-competent transgenic mice injected with CD8 T cells (Figure 6C). Note that nucleo-cytoplasmic translocation of HMGB-1 often reflects its release by necrotic, as opposed to apoptotic, cells [37]. The increased PMN recruitment in Clo-L treated mice occurred in face of intrahepatic CXCL1 and IL-17 (known PMN chemoattractants [38], [39]) expression levels that were comparable to those detected in control mice (Figure 2, G and H), which showed fewer cytoplasmic HMGB-1^+^ hepatocytes and fewer infiltrating PMNs (Figure 6C). Similar results were obtained in mice infected with Ad-β-Gal (not shown). Thus, impaired removal of apoptotic hepatocytes by KCs promoted accumulation of cytoplasmic HMGB-1^+^ hepatocytes and intrahepatic PMN infiltration. Recruited PMNs played a compensatory role in the removal of apoptotic hepatocytes from Clo-L-treated mice, as their depletion by anti-Gr-1 antibodies (Figure S6) was associated with detection of higher numbers of cytoplasmic HMGB-1^+^ hepatocytes (not shown) and higher sALT values (Figure 6D). Higher numbers of cytoplasmic HMGB-1^+^ hepatocytes (not shown), higher sALT values (Figure 6E) and reduced numbers of liver infiltrating PMNs (Figure S6) were also detected in Clo-L-treated HBV replication-competent transgenic mice that were administered with a blocking monoclonal Ab specific for mouse HMGB-1 (Figure S6) - prior to CD8 T cell transfer. Along with the finding that, when compared to controls, serum HMGB-1 levels were increased in Clo-L-treated mice that were injected with CD8 T cells or infected with Ad-β-Gal (not shown), these results reiterate the notion that the release of HMGB-1 from secondarily necrotic hepatocytes most likely contributed to recruit PMNs into the liver. Since PMN depletion and HMGB-1 neutralization prolonged disease severity in these animals only partially, we reasoned that, under the inflammatory conditions generated by CD8 T cells, the depleting effect of a single Clo-L administration (given 3 days prior to transfer) probably persisted less than one week (which is the time frame we and others have observed in un-manipulated mice, not shown and [24]). Indeed, F4/80^+^ KCs (likely derived from hematogenous monocyte precursors [40]) re-appeared in the liver 3 days after CD8 T cell transfer (Figure 7A), and a second injection of Clo-L at this time extended the local persistence of apoptotic and cytoplasmic HMGB-1^+^ hepatocytes (not shown) and the relative increase in sALT activity (Figure 7B) by about a week when compared to mice receiving a single dose of Clo-L (Figure 1A). Worsening of liver disease severity (and even mortality in the case of Clo-L treated mice) (Figure S7) was also observed in HBV replication-competent transgenic mice in which all WBC (including PMNs and monocytes) were completely eliminated by whole-body irradiation prior to CD8 T cell transfer (7), reiterating the notion that compensatory functions mediated by liver infiltrating phagocytic cells help KCs at containing liver immunopathology.

### The role of scavenger receptors

Although the administration of Clo-L- or GdCl3 in CD8 T cell-injected/Ad-β-Gal infected mice did not increase the liver expression of potentially hepatotoxic factors (e.g. IFN-γ, TNF-α or IL-17), it is formally possible that these treatments might have resulted in the release of undetermined cytotoxic factors exacerbating liver damage. To confirm that KCs contain liver immunopathology independently of Clo-L- or GdCl3 treatments and to provide mechanistic insight on how KCs phagocytose apoptotic hepatocytes, we made use of Polyinosinic acid (Poly(I)), a ligand for most macrophage scavenger receptors and a known blocker of KC phagocytosis [41]–[43]. Polyuridylic acid (Poly(U)), a non-scavenger receptor ligand, was used as control [41]–[43]. Of note, KCs isolated from the liver of HBV replication-competent or C57BL/6 mice were found to express macrophage scavenger receptor 1 (MSR-1) and scavenger receptor class b1 (Scarb-1) (Figure S8 and not shown), two prototypic class A and class B scavenger receptors, respectively, which can be bound by Poly(I) [41]–[43]. Neither Poly(I)- nor Poly(U)-treatment reduced the number of F4/80^+^ KCs (Figure S8) or the intrahepatic number of innate immune cells such as CD11c^high^ DCs, NK1.1^+^ CD3^-^ NK cells and NK1.1^+^ CD3^+^ NKT cells (Figure S8). Further, both of these treatments failed to reduce the number of circulating Gr-1^high^ CD11b^+^ PMNs (not shown). When compared to mice treated with Poly(U), however, mice treated with Poly(I) exhibited delayed removal of fluorescent microbeads from the circulation (Figure S8), providing evidence for the efficacy of this latter compound at inhibiting liver phagocytosis.

**Figure 8:**
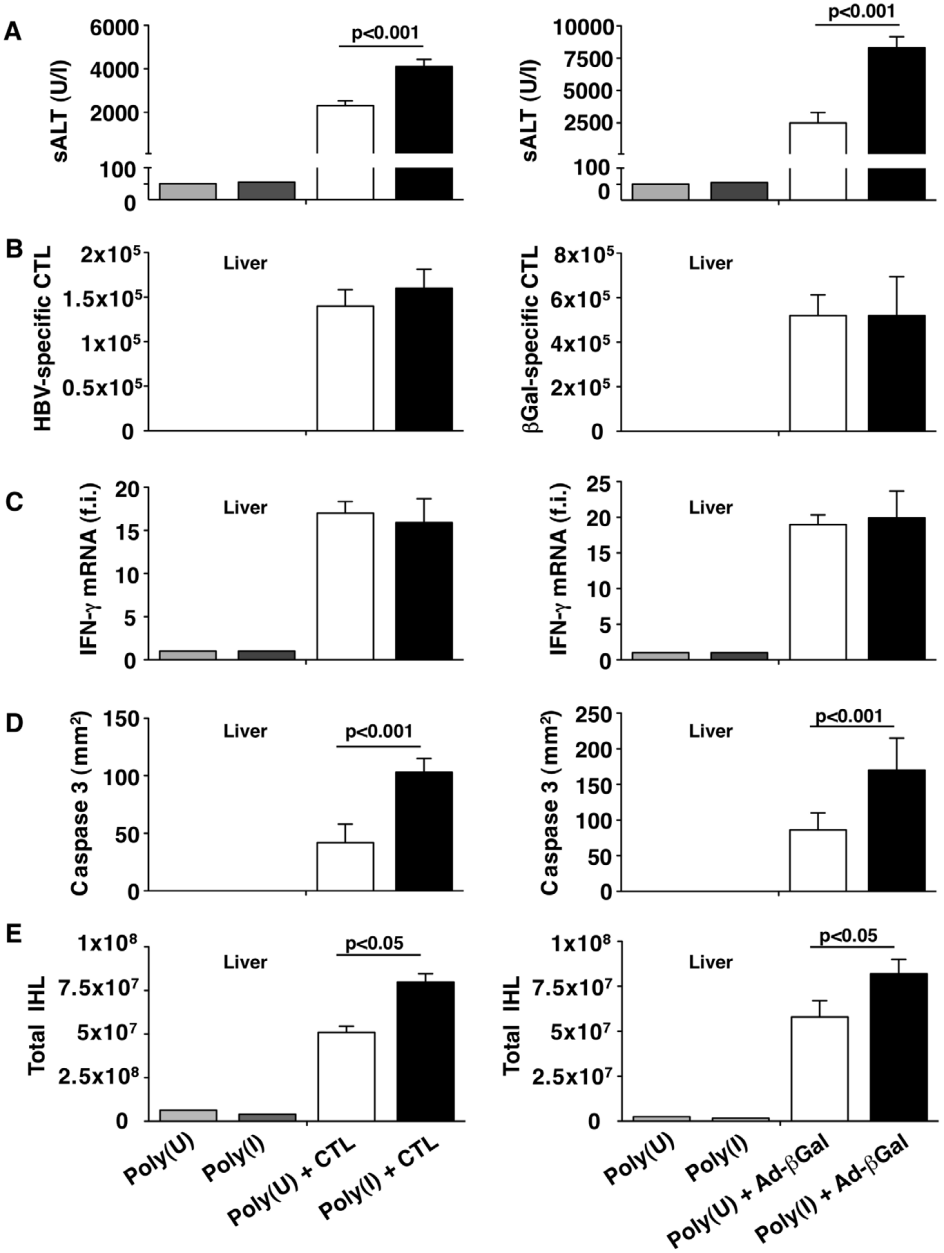
KCs remove apoptotic hepatocytes in a scavenger receptor-dependent manner. HBV replication-competent transgenic (left column) or wild-type (right column) mice were treated with the scavenger receptor inhibitor Poly(I) or its control Poly(U) prior to HBV-specific CTL transfer (in the case of HBV transgenic mice) or one day after Ad-β-Gal infection (for wild-type mice). All the analyses were performed at the peak of liver disease (two days after HBV-specific CTL transfer or four days after Ad-β-Gal infection. *n* = 6. (A) Mean sALT activity (units/liter); (B) absolute number of intrahepatic HBV-specific (left) or Ad-β-Gal-specific (right) CTL; (C) intrahepatic IFN-γ mRNA expression (assessed as in Figure 1G); (D) number of CC3^+^ hepatocytes (assessed as in Figure 4A and C) and (E) number of total intrahepatic leukocytes (IHL). All data are expressed as mean ± standard deviation and are representative of at least 3 independent experiments that gave similar results. Differences between Poly(I)- or Poly(U)-treated mice were not statistically significant unless otherwise indicated.

Importantly, HBV replication-competent transgenic mice and C57BL/6 mice that we treated with Poly(I) either 5 minutes before CD8 T cell transfer or 3 days after Ad-β-Gal infection (more than 2 days following maximal hepatocellular infection) displayed (at peak disease severity) higher sALT values than Poly(U)-treated controls (Figure 8A) and this was associated with i) similar numbers of intrahepatic virus-specific CD8 T cells (Figure 8B), ii) similar amounts of liver IFN-γ mRNA (Figure 8C), iii) increased accumulation of apoptotic (Figure 8D) and necrotic hepatocytes (not shown) and iv) increased infiltration of antigen non-specific leukocytes (Figure 8E). Together, our results, based on mechanistically distinct approaches, indicate that CD8 T cell-induced liver immunopathology can be worsened by depleting KCs or by inhibiting their scavenger receptor-dependent capacity to phagocytose apoptotic hepatocytes.

## Discussion

We show here that KCs play a previously unappreciated role in the pathogenesis of viral hepatitis, i.e. they contribute to the resolution of liver pathology induced by virus-specific effector CD8 T cells. We initially found that the injection of Clo-L (a treatment widely used to deplete murine KCs [23], [24]) was associated with a highly significant 2–3 fold increase in sALT activity (a commonly used marker of liver cell injury) at all time points measured. This was an unexpected finding since KCs are currently regarded as contributors to liver damage during viral hepatitis. Also unexpectedly we found that the overall number and function of intrahepatic virus-specific CD8 T cells - two factors directly linked to liver disease severity - were unaffected in these animals. Indeed, none of the various steps leading to hepatocellular destruction by CD8 T cells seemed impacted by the Clo-L treatment. The adhesive behavior of CD8 T cells to the liver microvasculature was shown to be normal, despite the fact that transient CD8 T cell/KC interactions often took place in the sinusoids of control mice. Clo-L treatment did not impinge on the capacity of CD8 T cells to recognize hepatocellular antigens either, as these cells produced IFN-γ (a cytokine that in our systems is exclusively expressed by *in vivo* activated virus-specific CD8 T cells [25]), divided and accumulated intrahepatically at control levels. Along with the notions that treatment with Clo-L alone did not induce liver inflammation and that the GdCl3-dependent depletion of KCs reproduced the CD8 T cell-dependent disease exacerbation observed after Clo-L treatment, these initial results indicated that KCs were not contributing to liver injury, either as effector cells directly involved in the destruction of hepatocytes, or as promoters of CD8 T cell-induced pathology.

The results also raised the question of how sALT levels in KC-depleted animals increased disproportionally when compared to the number or function of virus-specific effector CD8 T cells. Pertinent to this question it is worth mentioning that virus-specific effector CD8 T cells kill hepatocytes by apoptosis [44], [45]. Apoptotic hepatocytes preserve cell membrane integrity [46] and, as such, they do not release their cytosolic ALT content into the circulation. Accordingly, only necrotic hepatocytes should release ALT. As KCs are known to phagocytose apoptotic cells [47], we hypothesized that i) KC depletion resulted in the accumulation of apoptotic hepatocytes *in situ*, and that ii) these apoptotic cells secondarily evolved into ALT-releasing necrotic hepatocytes since they were not readily removed by KCs. Evidence supporting these hypotheses emerged from experiments where the administration of an inhibitor of KC phagocytosis that does not deplete macrophages also promoted high sALT levels, control-level intrahepatic numbers of pathogenic virus-specific CD8 T cells and, most relevantly, liver accumulation of apoptotic and necrotic hepatocytes. Notably, these experiments also identified scavenger receptors as mediators of KC-dependent phagocytosis of apoptotic hepatocytes during viral hepatitis. Additional evidence supporting the hypotheses emerged from histological analysis demonstrating progressive accumulation of hepatocellular apoptosis, hepatocellular necrosis and dropout in the liver of animals in which KCs were either reduced in their number or inhibited in their phagocytic function. These pathological signs were also accompanied by the detection of large numbers of hepatocytes displaying nucleo-cytoplasmic translocation of HMGB-1.

As nucleo-cytoplasmic translocation of HMGB-1 frequently reflects the release of this “danger signal” from necrotic cells [48] and as HMGB-1 release is known to trigger PMN liver recruitment [36], it was not surprising that PMNs were abundantly found in the liver of mice where KCs were numerically reduced or functionally inhibited. The fact that reduced PMN liver infiltration (mediated by either PMN depletion or HMGB-1 neutralization) worsened hepatic inflammation even further supports the concept that PMNs performed compensatory phagocytic functions in these livers. This is an interesting observation since PMNs have been previously reported to contribute to CD8 T-cell induced organ damage by facilitating the intrahepatic homing of antigen non-specific mononuclear cells [19], [20]. Thus, it would appear that HMGB-1-responding PMNs can exert more than just a detrimental role, since - like KCs - they have the potential to remove apoptotic hepatocytes and ameliorate liver immunopathology. It is relevant to point out, however, that this beneficial anti-inflammatory role of PMNs becomes apparent only under conditions of reduced KC function.

It is also relevant to point out that the experimental approaches we used to deplete KCs (intravenous injection of Clo-L or GdCl3) or to inhibit their function (intravenous injection of Poly(I)) are not exclusively specific for KCs (although they did not alter the frequency of circulating monocytes and PMNs). Indeed, these approaches have been shown to moderately impact other macrophage populations, particularly those (e.g. splenic mononuclear phagocytes) that - like KCs - are not separated from the bloodstream by an endothelial barrier [31], [32], [49]. Experiments utilizing splenectomized mice ruled out the possibility that splenic mononuclear phagocytes are involved in the exacerbation of liver disease severity observed in KC-depleted animals.

Of note, hepatic inflammation triggered by virus-specific effector CD8 T cells eventually subsided in mice treated with Clo-L, GdCl3 or Poly(I). This in part relates to the fact that viral antigens were rapidly eliminated from these livers, putting an early end to the CD8 T cell-induced pathology. Indeed, either HBV- or Ad-β-Gal-derived hepatocellular antigens disappeared within 2–3 days after peak disease severity [21] and remained down regulated for at least 4 weeks after transfer (in the case of HBV replication-competent transgenic mice), or they never returned (in the case of mice infected with the replication-deficient adenovirus) (not shown). Although significantly prolonged in its severity, hepatic inflammation eventually subsided even in animals subjected to multiple administrations of Clo-L (and multiple administrations of Poly(I), not shown). This reiterates the notion that compensatory phagocytic functions (likely mediated by PMNs and infiltrating monocytes with KC-precursor capacity) were operative under these conditions. Experiments utilizing mice in which all circulating WBC (including PMNs and monocytes) are permanently eliminated through whole-body irradiation further supported the “compensatory” concept. Predictably, restoration of anatomical integrity was accompanied by hepatocellular regeneration (evaluated by counting mitotic figures as well as the number of proliferating cell nuclear antigen [PCNA]- or Ki67-positive hepatocytes), which remained detectable until liver inflammation was completely resolved (not shown).

The results herein described contradict the current dogma that views KCs as solely pro-inflammatory cells during viral hepatitis. Indeed, the results indicate that, while KCs exhibit pro-inflammatory activities (such as the production of TNF-α), their overall net effect is anti-inflammatory. Our experiments were performed in animal models in which - as it occurs during HBV or HCV infection - virus-specific effector CD8 T cells that recognize hepatocellular antigens trigger viral hepatitis. While relevant to study the effector phase of liver immunopathology, our models were not designed to evaluate a possible role for KCs in the priming of virus-specific T cell responses. Future work will address this issue.

**Figure 9:**
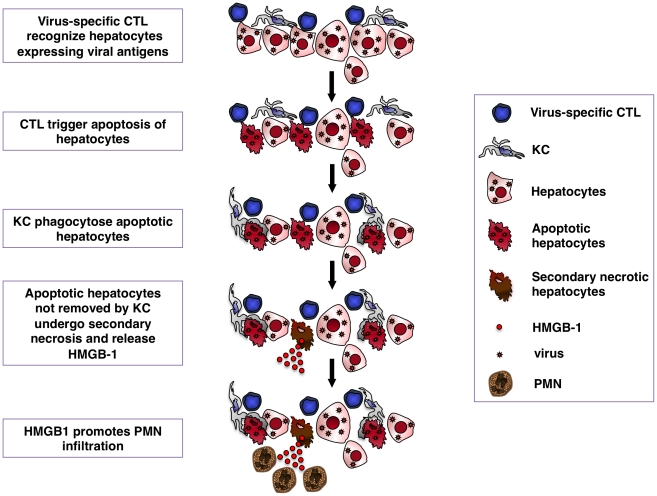
Schematic representation of the mechanisms whereby KCs contain CTL-induced liver pathology. Following antigen recognition virus-specific CTL trigger hepatocyte apoptosis. KCs readily remove apoptotic hepatocytes, thus limiting the release of HMGB-1 protein by secondary necrotic cells. HMGB-1 release promotes organ infiltration of inflammatory cells, particularly PMNs.

In conclusion, we found that KCs limit the severity of CD8 T cell-induced liver pathology in mouse models of viral hepatitis. Mechanistically, our data indicate that KCs limit liver immunopathology affecting neither the accumulation nor the function of intrahepatic virus-specific effector CD8 T cells with pathogenic potential. Rather, our results are most compatible with the hypothesis that KCs hasten resolution of liver immunopathology by removing apoptotic hepatocytes that are killed by effector CD8 T cells (see also the schematic representation depicted in Figure 9). Failure to do so results in the secondary necrosis of hepatocytes and abundant liver inflammation. Similar events may occur in humans during HBV and HCV infections, where CD8 T cell-dependent pathogenic mechanisms similar to those described herein are operative.

## Methods

### Ethics statement

This study was carried out in strict accordance with the recommendations in the Guide for the Care and Use of Laboratory Animals of the National Institutes of Health. These studies were approved by the Animal Review Board of the San Raffaele Scientific Institute (Permit Number 390) and by the Institutional Animal Committee of The Scripps Research Institute (Permit Number 09-0124). All surgery was performed with mice kept anesthetized by continuous administration of 2% isoflurane in 2 L/minute oxygen through a nose cone and all efforts were made to minimize suffering.

### Mice

HBV replication-competent transgenic mouse (lineage 1.3.32) have been previously described [16]. Lineage 1.3.32 (inbred C57BL/6, H-2^b^) was crossed with B10.D2 mice (H-2^d^) to produce H-2^bxd^ F_1_ hybrids prior to injection of H-2^d^-restricted hepatitis B surface antigen (HBsAg)-specific CD8 T cell lines. C57BL/6 and B6.PL-Thy1a/CyJ (Thy-1.1) mice were purchased from The Scripps Research Institute breeding colony or from Charles River Laboratories (Calco, Italy). Bone marrow chimeric phosphoglycerate kinase (PGK)-GFP mice replicating HBV were created by transplanting BM cells derived from PGK-GFP (H-2^bxd^ F_1_ hybrids, a kind gift of Michele De Palma, San Raffaele Scientific Institute, Milan, Italy) into irradiated 1.3.32 HBV mice. Thy-1.1 mice were crossed once with B10.D2 mice prior to immunization with plasmid DNA- and vaccinia virus-encoding HBsAg as previously described [21]. In some experiments, mice were subjected to whole-body irradiation or splenectomy, as described [50], [51]. In all experiments mice were matched for age (8 weeks), sex (males) and, in case of lineage 1.3.32, for serum hepatitis B e antigen (HBeAg) levels before experimental manipulation. All animals were housed in pathogen-free rooms under strict barrier conditions.

### Injection of HBV-specific CD8 T cell lines

HBV-specific CD8 T cell lines were derived from spleen cells of immunized nontransgenic Thy-1.1 x B10.D2 male mice as described [21]. After 3 weeks of *in vitro* stimulation, the cells were tested for antigen specificity by flow cytometry as described [18], [21]. CD8^+^ cells that were over 95% specific for the immunodominant peptide epitope Env 28–39 of HBsAg [44] were injected intravenously at different doses (0.5×10^7^ cells/mouse, 1×10^7^ cells/mouse or 5×10^7^ cells/mouse) into 1.3.32 mice. One, 2, 3, 5, 7 or 14 days later mice were killed and their livers were perfused and harvested for histological and flow cytometry analyses, or they were snap frozen in liquid nitrogen and stored at -80°C for subsequent molecular analyses (see below).

### β-Gal DNA immunization and adenovirus infection

Fifty micrograms of a plasmid expressing β-Gal under the control of the human CMV enhancer/promoter were injected into regenerating tibialis anterior muscles of C57BL/6 mice 5 days after injection of cardiotoxin as described [21]. Three weeks later mice were grouped based on the frequency of circulating CD8^+^/β-Gal96^+^ T cells (between 0.2% and 0.3% of the total white blood cells) and infected with a single intravenous dose (1×10^9^ pfu/mouse) of a β-Gal-expressing adenovirus vector (Ad-β-Gal) as described [21]. Mice were killed 1, 3, 4, 5 and 7 days after infection and their livers were processed as described above. The immunization strategy abovementioned allowed us to focus our attention on CD8 T cell effector functions that are independent of priming, and to quantitatively measure the β-Gal-specific CD8 T cell response. Indeed, under these conditions mice develop a severe liver injury that is entirely mediated by an effector memory CD8 T cell response specific for a single H2^b^-restricted immunodominant epitope (β-Gal96) contained within β-Gal. The response precedes any other adenovirus-specific CTL response [21].

### Depletion of KCs and PMNs

KC depletion was achieved by intravenous injection of 200 µl of clodronate-containing liposomes (Clo-L, a gift of Roche Diagnostics GmbH, Mannheim, Germany) 3 days before CD8 T cell transfer or one day after Ad-β-Gal infection. In some experiments saline-containing liposomes (NaCl-L) were used as control. In other experiments KCs were depleted by the intravenous injection of 50 µg of gadolinium chloride (GdCl3) 24 hours and 30 minutes before CD8 T cell transfer. In selected experiments a second Clo-L injection was administered into 1.3.32 mice 3 days after CD8 T cell transfer. PMN depletion was achieved by intravenous injection of 100 µg of a rat IgG2b monoclonal antibody specific for mouse Gr-1 (Ly-6G/Ly-6C, clone RB6-8C5; BD PharMingen) on days 1, 2 and 3 after CD8 T cell transfer as described [19]. Control mice received an equal volume of a rat IgG2b irrelevant (Irr) Abs (clone A95-1; BD PharMingen) at the same time points. Although clone RB6-8C5 has been shown to deplete subsets of dendritic cells and monocytes [52], [53], it has no effect on KCs. Indeed, we detected comparable numbers of liver F4-80^+^ cells in mice injected with either RB6-8C5 or the IgG2b control (not shown).

### Generation, characterization and in vivo administration of α-HMGB-1 Ab (DPH1.1 Ab)

The mouse monoclonal IgG1 DPH1.1 Ab specific for mouse HMGB-1 was generated by injecting C57BL/6 mice at two-week intervals with four doses (50 mg/mouse) of the 17-mer peptide P1 (KGKPDAAKKGVVKAEKS) derived from HMGB-1. Hybridomas were generated from splenocytes by standard techniques and tested by ELISA against the immunogen. Specificity of DPH1.1 Ab was monitored by both Western blot and immunofluorescence as shown in Figure S4. Briefly, 500 ng or 100 ng of recombinant HMGB-1 and the (negative control) recombinant Box-A fragment of HMGB-1 (HMGBiotech, Milan, Italy) were separated by gel electrophoresis and transferred onto membranes as described [48]. DPH1.1 Ab, anti-Box-A Ab (HMGBiotech, Milan, Italy) and goat anti-mouse IgG1 Ab (BD PharMingen) were applied at 1 µg/ml dilution. Immunofluorescence was performed as described [48] on mouse embryonic fibroblasts (MEFs) derived from either wild type mice or *HMGB-1* ^−/−^ mice. DPH1.1 Ab and AlexaFluor 633-labelled goat anti-mouse IgG1 Ab (BD PharMingen) were applied at a 50-µg/ml dilution. The i*n vitro* activity of DPH1.1 Ab was monitored in trans-well migration assays as basically described [54] and shown in Figure S4. Briefly, 3T3 cells were assessed for their migration ability by a modified Boyden chamber assay. Recombinant HMGB-1 was added to the lower chamber at the concentration of 30 ng/ml. Increasing concentrations of DPH1.1. Ab were added to fifty thousand 3T3 cells seeded in the upper chamber. Boyden chambers were incubated at 37°C in 5% CO_2_ for 3 hours. Cells remaining on the upper section of the filters were removed mechanically. Cells that migrated to the lower section of the filters were fixed with ethanol, stained with Giemsa (Sigma-Aldrich), and counted in 10 random fields/filter. Each assay was performed in triplicate and repeated at least three times, independently. *In vivo,* DPH1.1. Ab was administered intravenously (220 µg/mouse) 3 hours before CD8 T cell transfer. Control mice received an equal amount of a mouse IgG1 control Abs (BD PharMingen) at the same time point.

### Analysis of KC phagocytic functions

Clo-L- or Poly(I)-treated mice were intravenously injected with 5×10^8^ rhodamine beads of 4 µm in diameter (provided by Z. M. Ruggeri, The Scripps Research Institute, La Jolla, CA). Immediately after injection and 2, 4, 6, 10, 15, 20, 25 and 30 minutes later mice were bled and the number of beads in blood samples was assessed by flow cytometry. Livers from selected mice were processed by confocal microscopy as described below.

### Liver intravital microscopy

PGK-GFP-1.3.32 BM chimeras and 1.3.32 mice treated or not with Clo-L were kept anesthetized by continuous administration of 2% isoflurane (Abbott S.r.l, Aprilia, Italy) in 2 L/minute oxygen through a nose cone. After the insertion of a tail vein polyethylene catheter attached to a syringe-pump able to deliver continuous infusion of a 37°C saline solution (0.25 ml/hr), mice underwent surgery. After opening the skin with a midline incision and detaching peritoneal adherences, midline and left subcostal incisions were made in the peritoneum through a high-temperature cautery. The left liver lobe was exteriorized and placed within a U-shaped, water-holding, silicon chamber placed on an adjustable thin base. The chamber was then covered with a cover slip at the bottom of which the left liver lobe gently flattened. The stage was then moved to a heated microscope stage of an up-right Axiotech Vario microscope equipped with a Colibri system of high-performance Light Emitting Diodes (LEDs) that are fully integrated/automated by AxioVision system software (Carl Zeiss, Göttingen, Germany), allowing high contrast images with simultaneous 3 color-imaging in real time. HBV-specific effector CD8 T cells were fluorescently labeled with either CFSE (20 µM for 7 minutes at room temperature) or Hoechst 33342 (2 µg/ml for 15 minutes at 37°C; Invitrogen, Carlsbad, CA). Importantly, CFSE- or Hoechst-labeled HBV-specific CD8 T cells caused the same sALT elevation as unlabeled cells upon *in vivo* transfer (not shown). Control effector CD8 T cells (specific for the lymphocytic choriomeningitis virus, LCMV) were derived from the spleen of mice (C57BL/6 × B10 D2 F_1_) that resolved an acute LCMV infection and were *in vitro* stimulated as described [55]. Labeled CD8 T cells (1 or 5×0^7^ cells/mouse) were transferred into mice through the tail vein catheter and parameters of cell motility and adhesion to liver vasculature were recorded with a AxioCam HSC color videocamera (Carl Zeiss, Göttingen, Germany) at an acquisition rate of 15 frames/second. The sticking fraction of HBV-specific CD8 T cells was defined as the percentage of total cells that became firmly adherent for ≥30 s while passing a liver sinusoid within a 30 minutes observation period, as described [56].

### Injection of liver extracts, Poly(I) and Poly(U)

Liver extracts containing ∼6000 U of ALT were prepared as previously described [17] and injected into 1.3.32 mice. Poly(I) and its control Poly(U) were injected intravenously (200 µg/mouse, Sigma) into 1.3.32 mice or C57BL/6 mice 5 minutes before CD8 T cell transfer or 3 days after Ad-β-Gal infection, respectively.

### Analyses of intrahepatic cell subsets

Intrahepatic leukocyte (IHL) isolation was performed as described [20], [21]. Cells were surface-stained with phycoerythrin (PE)-conjugated anti-CD4 (clone RM4-5; BD Pharmingen) and anti-CD11c (clone HL3; BD Pharmingen); Pacific Blue-conjugated anti-CD8 (clone 53-6.7; BD Pharmingen) and anti-CD3 (clone 145-2c11; BD Pharmingen); PE-Cy7-conjugated anti-CD11b (clone M1/70; BD Pharmingen); allophycocyanin (APC)-conjugated anti-TCR (clone H57-597; BD Pharmingen) and anti-Ly6G (clone 1A8; BD Pharmingen); fluorescein isothiocyanate (FITC)-conjugated anti-Gr-1 (clone RB6-8C5; BD Pharmingen). HBV-specific CD8 T cells were quantified by staining IHL with PE-conjugated anti-Thy1.1 (clone OX7; BD Pharmingen) and APC-conjugated anti-TCR (clone H57-597; BD Pharmingen) as described [20], [21]. Ad-β-Gal-specific CD8 T cells were quanftified from PBMC or IHL by intracellular IFN-γ staining using a recombinant soluble dimeric H-2K^b^:Ig Fusion Protein (BD Pharmingen) complexed with the β-Gal96 immunodominant peptide as described [21]. Liver non-parenchymal cells enriched of KCs were isolated as previously described [57].

### DNA and RNA analyses

Total DNA and RNA were isolated from blood or frozen livers (left lobe) for analyses by Southern blot, Northern blot, RNAse protection (RPA) and real-time PCR as previously described [17], [55]. Nylon membranes were analyzed for HBV DNA, β-Gal DNA and RNA, and glyceraldehydes-3-phosphate dehydrogenase (GAPDH) RNA as previously described [25]. Analysis of cytokine, chemokine and scavenger receptors mRNAs was performed by RPA or TaqMan Gene expression Assay (Applied Biosystems) as previously described [17], [19], [20]. Real-time PCR for Ad-β-Gal was performed as described [21].

### Liver disease

The extent of hepatocellular injury was monitored by histological analysis and by measuring sALT activity as described [17]. Quantitative morphometry was carried out by calculating the mean size of necroinflammatory foci contained in 100 high power fields, corresponding to about 4 mm^2^ of liver tissue, as described [21].

### Immunohistochemistry, immunofluorescence and confocal microscopy

Immunohistochemical staining for hepatitis B core antigen (HBcAg), HMGB-1, CC3, PCNA and Ki67 was performed as described [16], [17], [36], [58]. The number of CC3^+^ hepatocytes was calculated in 100 high power fields, corresponding to about 4 mm^2^ of liver tissue. Immunofluorescence staining for F4/80 was performed as described [50]. Confocal microscopy was carried out with an Axioskop 2 plus direct microscope (Zeiss) equipped with a Radiance 2100 three-laser confocal device (Bio-Rad). Images were analyzed with Paint Shop Pro X (Corel).

### Serum HMGB-1 ELISA

The serum concentration of HMGB-1 was measured by the use of the mouse ELISA kit II (Shino-Test Corporation, Japan) as previously described [59].

### Statistical analysis

In all studies, values are expressed as mean ± SD. All statistical analyses were performed in Prism (GraphPad Software). Means between two groups were compared by using a two-tailed t-test. Means between three or more groups were compared by using a one-way or two-way analysis of variance with Bonferroni's post-test. Kaplan–Meier survival curves were compared by using the log-rank (Mantel–Cox) test. Differences were considered statistically significant at p<0.05.

### Accession numbers mentioned in the text

The GeneBank or NCBI Reference Sequence (RefSec) numbers for the genes and proteins cited in the text are: IFN-γ K00083.1 (GeneBank); CXCL9, NC_000071.5 (RefSec); CXCL10, NC_000071.5 (RefSec); TNF-α, M11731.1 (GeneBank); IL-10, M37897.1 (GeneBank); TGF-β M13177.1 (GeneBank); CXCL1, NC_000071.5 (RefSec); HMGB-1, NC_000013.10 (RefSec); MSR-1 NM_031195.2 (RefSeq); Scarb-1 NM_016741.1 (RefSeq).

## Supporting Information

Figure S1 The effect of Clo-L- or GdCl3-treatments. (A) Representative confocal micrographs of control (left panel) or Clo-L-injected (right panel) HBV replication-competent transgenic livers, three days after treatment. Anti-F4/80 staining in red, TO-PRO-3 (TP3) staining of nuclei in blue. Scale bar represents 150 µm. *n* = 3. (B) Absolute number of intrahepatic CD11c^high^ DCs recovered from the mice described in (A). *n* = 3. (C) Frequency of blood Gr-1^high^ CD11b^+^ PMNs and (D) Ly-6C^+^ monocytes recovered from the mice described in (A). *n* = 3. (E) Representative confocal micrograph of HBV replication-competent transgenic livers 5 minutes after intravenous injection of red fluorescent beads. Anti-F4/80 staining in blue. Scale bar represents 10 µm. *n* = 3. (F) Bead concentration in the blood of control (NaCl) or Clo-L-treated HBV replication-competent transgenic mice. *n* = 6. (G) Representative confocal micrographs of control (left panel) or GdCl3-injected (right panel) HBV replication-competent transgenic livers, one day after treatment cessation. Anti-F4/80 staining in red, TO-PRO-3 (TP3) staining of nuclei in blue. Scale bar represents 150 µm. *n* = 3. All data are expressed as mean ± standard deviation and are representative of at least 3 independent experiments that gave similar results; differences between mice treated or not with Clo-L were not statistically significant unless otherwise indicated, * *p*<0.05, ** *p*<0.001.
(TIF)

Figure S2 Similar liver disease severity in mice administered with either saline (NaCl) or saline-containing liposomes (NaCl-L) prior to CTL transfer. Mean sALT activity (units/liter) measured at the indicated time points after intravenous injection of 10^7^ HBV-specific CTL in HBV replication-competent transgenic mice that received the indicated treatment (NaCl + CTL or Clo-L + CTL mice representing additional controls of this specific experiment have been described in Figure 3A). *n* = 6. Data are expressed as mean ± standard deviation and are representative of at least 2 independent experiments that gave similar results; note that no difference in sALT activity was detected between NaCl- and NaCl-L-injected mice at all time points after CTL transfer.
(TIF)

Figure S3 Clo-L treatment does not affect sALT half-life. Mean sALT activity (units/liter) measured at the indicated time points after intravenous injection of liver extracts of a known ALT content (∼6000 U) in HBV replication-competent transgenic control (white) or Clo-L-treated (black) mice. *n* = 6. All data are expressed as mean ± standard deviation and are representative of at least 3 independent experiments that gave similar results. Differences were not statistically significant.
(TIF)

Figure S4 Clo-L treatment does not affect HBcAg clearance. Representative immunohistochemical micrographs of HBV replication-competent transgenic livers five days after intravenous injection of NaCl (left panel), NaCl +10^7^ HBV-specific CTL (middle panel) or Clo-L +10^7^ HBV-specific CTL. HBcAg staining in brown. Scale bar represents 150 µm.
(TIF)

Figure S5 Comparable serum levels of albumin or bilirubin in mice treated or not with Clo-L. Mean serum levels of albumin (A) or bilirubin (B) measured at the indicated time points after intravenous injection of 10^7^ HBV-specific CTL in HBV replication-competent transgenic mice that received the indicated treatment. *n* = 6. All data are expressed as mean ± standard deviation and are representative of at least 3 independent experiments that gave similar results. Differences were not statistically significant.
(TIF)

Figure S6 α-Gr-1 and α-HMGB-1 Abs reduce blood and liver PMNs counts. (A) Absolute number of Gr-1^high^ CD11b^+^ PMNs in the blood of HBV replication-competent transgenic CTL-injected mice that received NaCl (white squares), Clo-L and irrelevant (Irr) Abs (black triangles) or Clo-L and anti-Gr-1 antibodies (αGr-1, black circles). *n* = 6. (B) Absolute number of Gr-1^high^ CD11b^+^ neutrophils (PMNs) recovered from the livers of mice that received NaCl (white squares), Clo-L and irrelevant (Irr) Abs (black triangles) or Clo-L and the anti-HMGB-1 Ab DPH1.1 (αHMGB-1, black circles) along with the intravenous injection of 10^7^ HBV-specific CTL. (C) Representative western blots of 500 ng (lane 1) or 100 ng (lane 2) of the recombinant Box-A fragment of HMGB-1 (negative control) and 500 ng (lane 3) or 100 ng (lane 4) of recombinant HMGB-1 incubated with either the α-HMGB-1 Ab DPH1.1. (top panel) or a control anti-Box-A fragment Ab control (bottom panel). (D) Representative micrographs of mouse embryonic fibroblasts (MEFs) derived from either wild type (wt) mice or HMGB-1^−/−^ mice that were stained by immunofluorescence with the α-HMGB-1 Ab DPH1.1. Scale bar represents 20 µm. (E) Transmigration of 3T3 cells toward a HMGB-1 gradient in the presence of the indicated concentrations of the α-HMGB-1 Ab DPH1.1 was examined using a modified Boyden chamber assay. Each bar represents the mean number of migrated cells ± standard deviation of triplicate samples.
(TIF)

Figure S7 The effect of whole-body irradiation on liver disease severity. Clo-L treated and relative control HBV replication-competent transgenic mice were subjected to whole-body irradiation four hours prior to the transfer of 10^7^ HBV-specific CTL. (A) Frequency of blood Gr-1^high^ CD11b^+^ polymorphonuclear neutrophils (PMNs) and (B) Ly-6C^+^ monocytes recovered four hours after whole-body irradiation. *n* = 3. (C) Mean sALT activity (units/liter) measured at the indicated time points after CTL transfer in mice that received the indicated treatment. *n* = 6. (D) Kaplan-Meier survival curves of control (white) or Clo-L-treated (black) whole-body irradiated mice described in (B). *n* = 6. (E) Representative micrographs of hematoxylin/eosin-stained of Clo-L + CTL- (left), Irradiation + NaCl + CTL- (middle) or Irradiation + Clo-L + CTL- (right) treated HBV replication-competent transgenic livers, three days after intravenous injection of 10^7^ HBV-specific CTL. Broken line delineates necroinflammatory foci. Note the abundant inflammatory infiltrate in the liver of mice treated with Clo-L + CTL (left), as opposed to what detected in the liver of mice treated with Irradiation + Clo-L + CTL (right). Note also the lack of large areas of damaged hepatocytes (and abundant inflammatory infiltrates) in irradiated mice not treated with Clo-L. All data are expressed as mean ± standard deviation and are representative of at least 2 independent experiments that gave similar results; differences between CTL-injected mice treated or not with Clo-L were not statistically significant unless otherwise indicated, ** *p*<0.001.
(TIF)

Figure S8 The scavenger receptor ligand Poly(I) inhibits liver phagocytosis. (A) Total RNA isolated from HBV-specific CTL, total liver RNA or KCs were analyzed by real time qPCR for the expression of macrophage scavenger receptor 1 (MSR-1) and scavenger receptor class b1 (Scarb-1). Results are expressed as arbitrary units after normalization for the housekeeping gene GAPDH. *n* = 6. (B) Representative confocal micrographs of HBV replication-competent transgenic livers 5 minutes after intravenous injection of Poly(U) (top panel) or Poly(I) (bottom panel). Anti-F4/80 staining in red, TO-PRO-3 (TP3) staining of nuclei in blue. Scale bar represents 150 µm. *n* = 3. (C) Absolute number of intrahepatic CD11c^high^ DCs, NK1.1^+^ CD3^−^ NK cells and NK1.1^+^ CD3^+^ NKT cells recovered from the mice described in (B). *n* = 3. (D) Bead concentration in the blood of HBV replication-competent transgenic mice that received the indicated treatment. *n* = 6. All data are expressed as mean ± standard deviation and are representative of at least 3 independent experiments that gave similar results; differences between mice treated with Poly(U) or Poly(I) were not statistically significant unless otherwise indicated; * *p*<0.05.
(TIF)

Video S1 PGK-GFP 1.3.32 HBV chimeric mice in which KCs and circulating white blood cells express GFP (green) were subjected to liver intravital microscopy. The movie depicts periportal liver sinusoids in a representative mouse that was infused 15 minutes earlier with 10^8^ rhodamine-labeled beads (red) and 5 minutes earlier with 10^6^ Hoechst-labeled HBV-specific effector CD8 T cells (blue). The movie shows an example of a virus-specific effector CD8 T cell arresting in close proximity and transiently coming in contact with a KC. Analysis of 30 minutes movies derived from 6 independent chimeric mice showed 30% of visualized HBV-specific effector CD8 T cells interacting with KCs (mean interaction time of 5±1 second). Scale bar represents 50 µm.
(MOV)

Video S2 HBV replication-competent transgenic mice were injected with NaCl and 3 days later subjected to liver intravital microscopy. The movie depicts lobular liver sinusoids in a representative mouse that was infused 5 minutes earlier with 5×10^7^ CFSE-labeled HBV-specific effector CD8 T cells. Analysis of movies derived from six independent HBV replication-competent transgenic mice revealed a sticking fraction (defined as the percentage of total CD8 T cells that became firmly adherent for ≥30 s while passing a liver sinusoid within a 30 minutes observation period) of 29.2%±3.1%. Note that the sticking fraction of effector CD8 T cells that were specific for an irrelevant antigen (LCMV) and were infused into HBV replication-competent transgenic mice was detected as less than 5% (not shown). Scale bar represents 50 µm.
(MOV)

Video S3 HBV replication-competent transgenic mice were injected with Clo-L and 3 days later subjected to liver intravital microscopy. The movie depicts lobular liver sinusoids in a representative mouse that was infused 5 minutes earlier with 5×10^7^ CFSE-labeled HBV-specific effector CD8 T cells (green). Analysis of movies derived from six independent HBV replication-competent transgenic mice revealed a sticking fraction (defined as the percentage of total CD8 T cells that became firmly adherent for ≥30 s while passing a liver sinusoid within a 30 minutes observation period) of 27.1%±1.9%. Scale bar represents 50 µm.
(MOV)
