# Supplementary figures and images for: Kupffer Cells Hasten Resolution of Liver Immunopathology in Mouse Models of Viral Hepatitis
Source: PLoS Pathog. 2011 Jun 2;7(6):e1002061. doi: 10.1371/journal.ppat.1002061 (PMC3107209; doi:10.1371/journal.ppat.1002061)

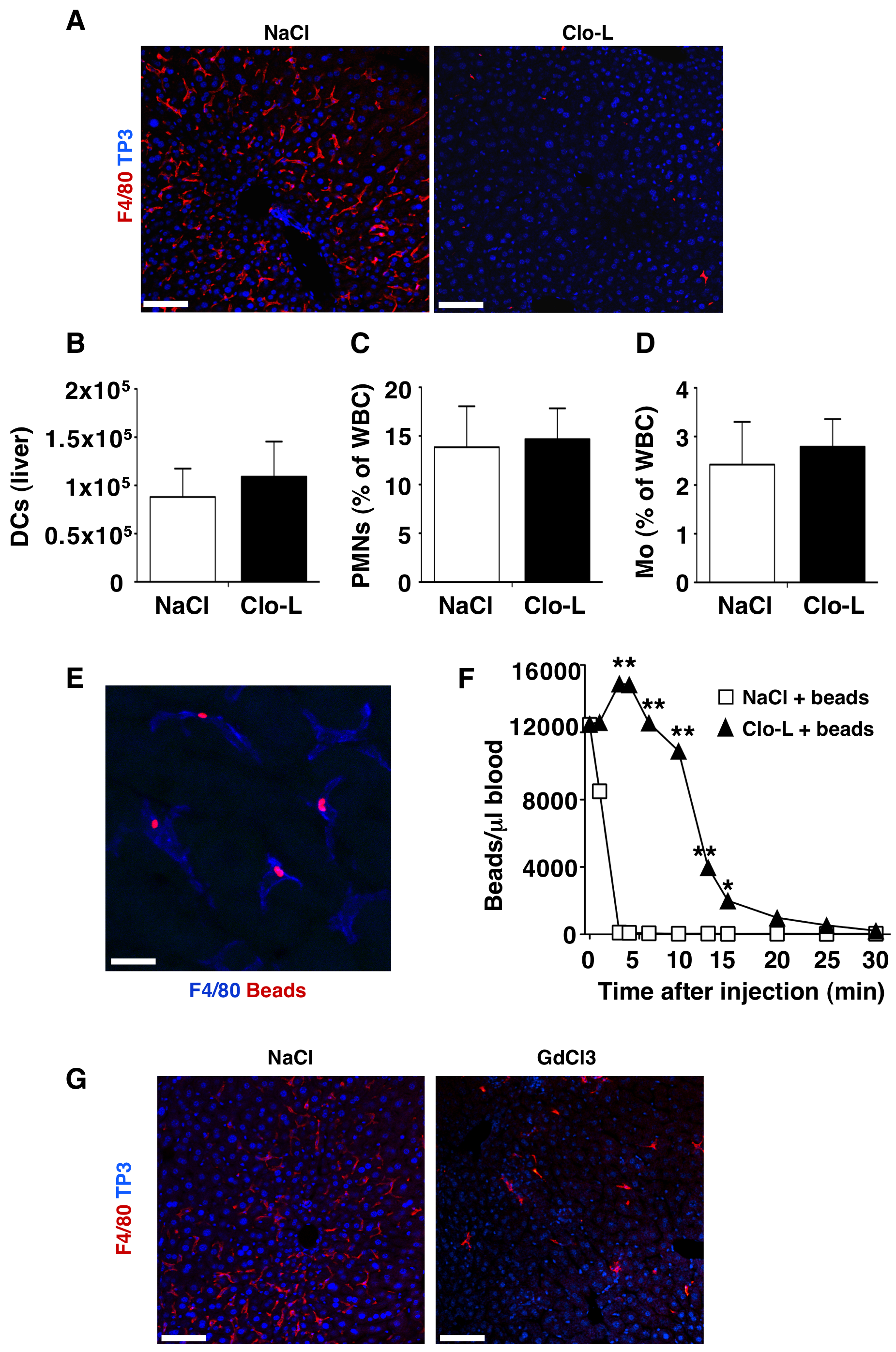

Supplement: Figure S1 — The effect of Clo-L- or GdCl3-treatments. (A) Representative confocal micrographs of control (left panel) or Clo-L-injected (right panel) HBV replication-competent transgenic livers, three days after treatment. Anti-F4/80 staining in red, TO-PRO-3 (TP3) staining of nuclei in blue. Scale bar represents 150 µm. n = 3. (B) Absolute number of intrahepatic CD11chigh DCs recovered from the mice described in (A). n = 3. (C) Frequency of blood Gr-1high CD11b+ PMNs and (D) Ly-6C+ monocytes recovered from the mice described in (A). n = 3. (E) Representative confocal micrograph of HBV replication-competent transgenic livers 5 minutes after intravenous injection of red fluorescent beads. Anti-F4/80 staining in blue. Scale bar represents 10 µm. n = 3. (F) Bead concentration in the blood of control (NaCl) or Clo-L-treated HBV replication-competent transgenic mice. n = 6. (G) Representative confocal micrographs of control (left panel) or GdCl3-injected (right panel) HBV replication-competent transgenic livers, one day after treatment cessation. Anti-F4/80 staining in red, TO-PRO-3 (TP3) staining of nuclei in blue. Scale bar represents 150 µm. n = 3. All data are expressed as mean ± standard deviation and are representative of at least 3 independent experiments that gave similar results; differences between mice treated or not with Clo-L were not statistically significant unless otherwise indicated, * p<0.05, ** p<0.001. (TIF) [file ppat.1002061.s001.tif]

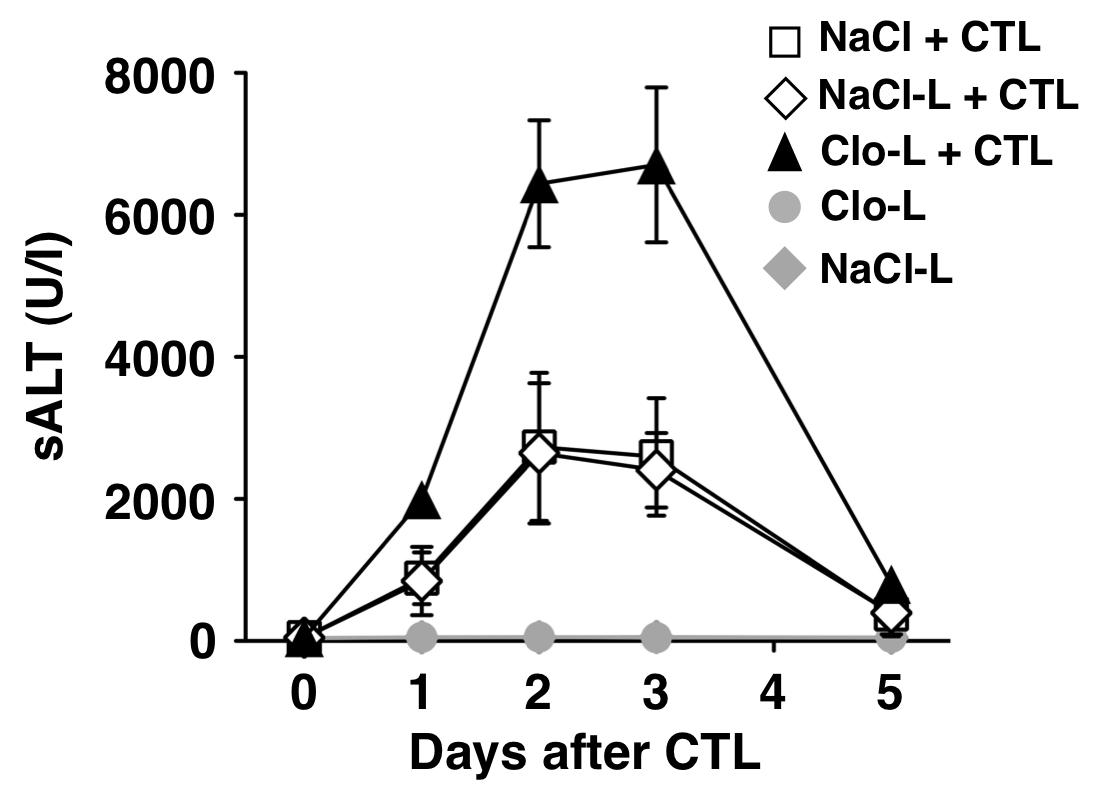

Supplement: Figure S2 — Similar liver disease severity in mice administered with either saline (NaCl) or saline-containing liposomes (NaCl-L) prior to CTL transfer. Mean sALT activity (units/liter) measured at the indicated time points after intravenous injection of 107 HBV-specific CTL in HBV replication-competent transgenic mice that received the indicated treatment (NaCl + CTL or Clo-L + CTL mice representing additional controls of this specific experiment have been described in Figure 3A). n = 6. Data are expressed as mean ± standard deviation and are representative of at least 2 independent experiments that gave similar results; note that no difference in sALT activity was detected between NaCl- and NaCl-L-injected mice at all time points after CTL transfer. (TIF) [file ppat.1002061.s002.tif]

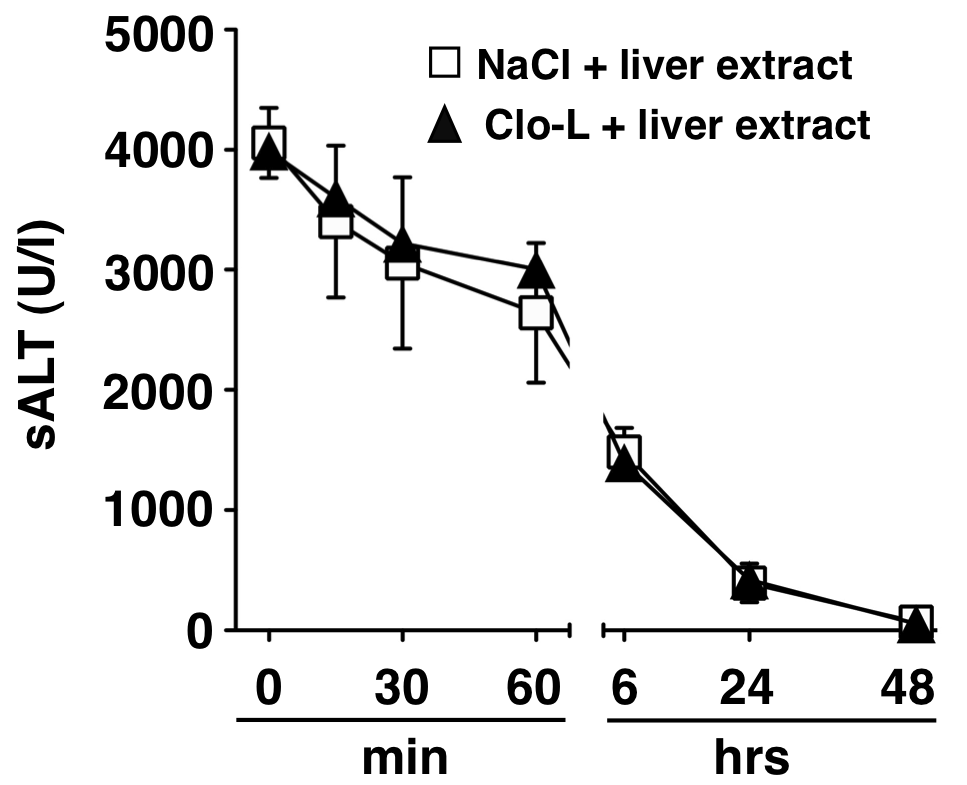

Supplement: Figure S3 — Clo-L treatment does not affect sALT half-life. Mean sALT activity (units/liter) measured at the indicated time points after intravenous injection of liver extracts of a known ALT content (∼6000 U) in HBV replication-competent transgenic control (white) or Clo-L-treated (black) mice. n = 6. All data are expressed as mean ± standard deviation and are representative of at least 3 independent experiments that gave similar results. Differences were not statistically significant. (TIF) [file ppat.1002061.s003.tif]

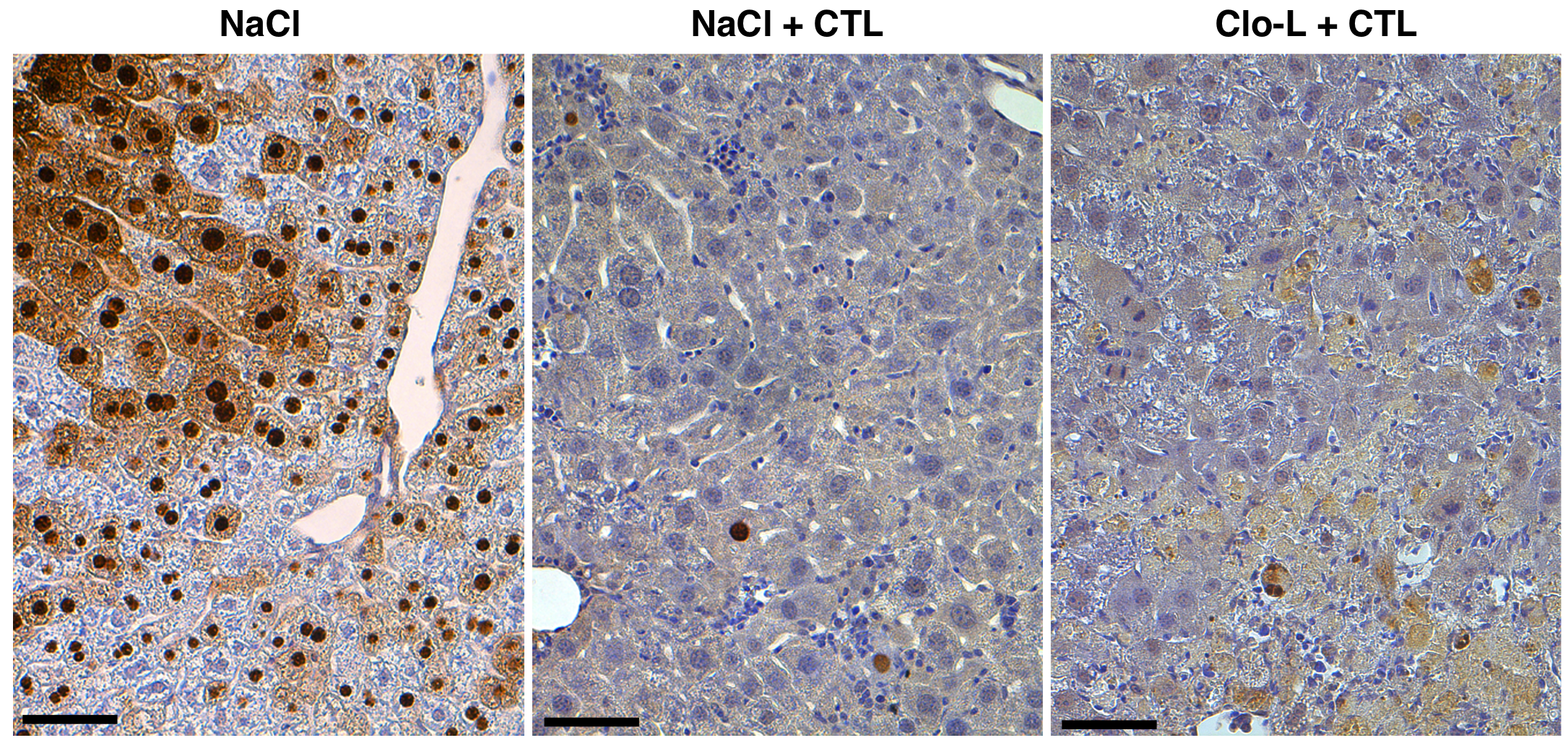

Supplement: Figure S4 — Clo-L treatment does not affect HBcAg clearance. Representative immunohistochemical micrographs of HBV replication-competent transgenic livers five days after intravenous injection of NaCl (left panel), NaCl +107 HBV-specific CTL (middle panel) or Clo-L +107 HBV-specific CTL. HBcAg staining in brown. Scale bar represents 150 µm. (TIF) [file ppat.1002061.s004.tif]

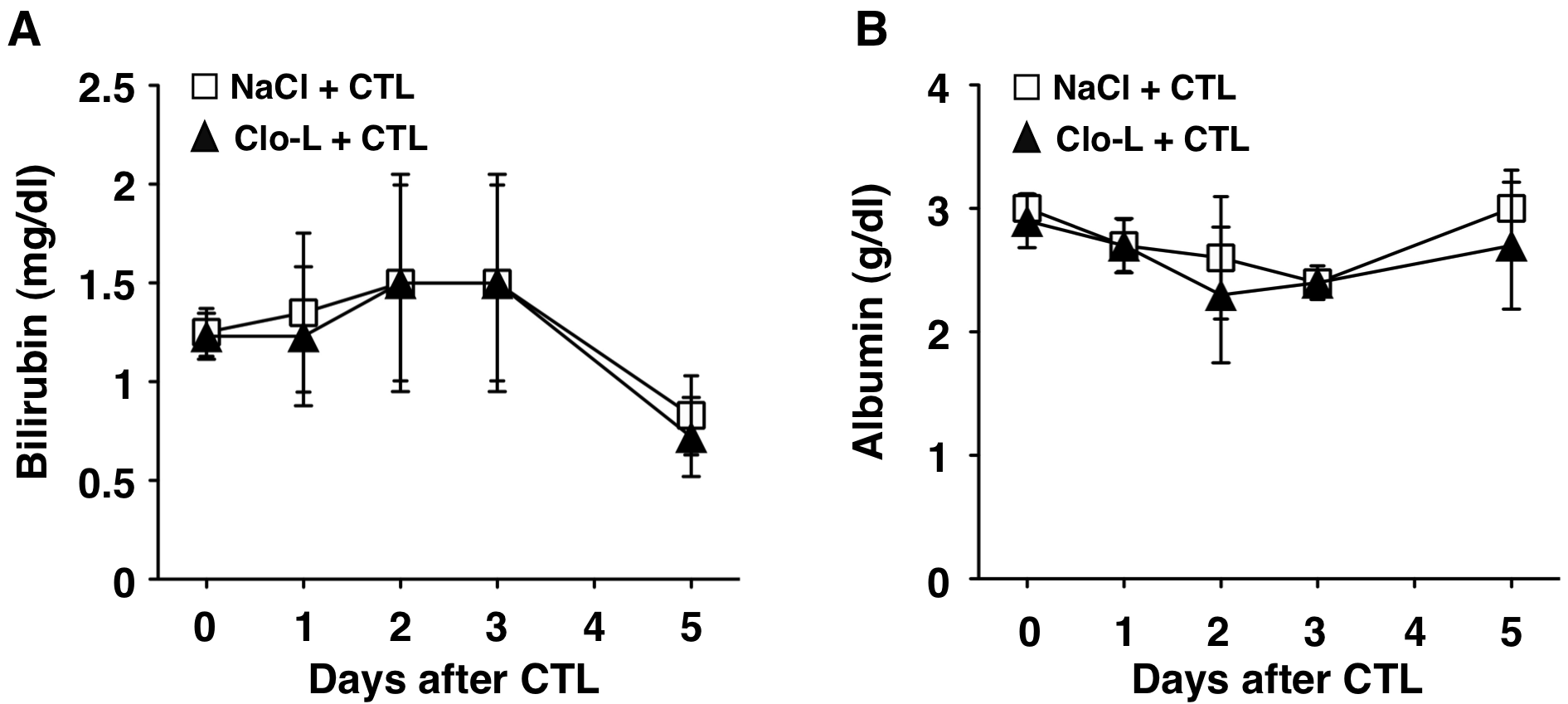

Supplement: Figure S5 — Comparable serum levels of albumin or bilirubin in mice treated or not with Clo-L. Mean serum levels of albumin (A) or bilirubin (B) measured at the indicated time points after intravenous injection of 107 HBV-specific CTL in HBV replication-competent transgenic mice that received the indicated treatment. n = 6. All data are expressed as mean ± standard deviation and are representative of at least 3 independent experiments that gave similar results. Differences were not statistically significant. (TIF) [file ppat.1002061.s005.tif]

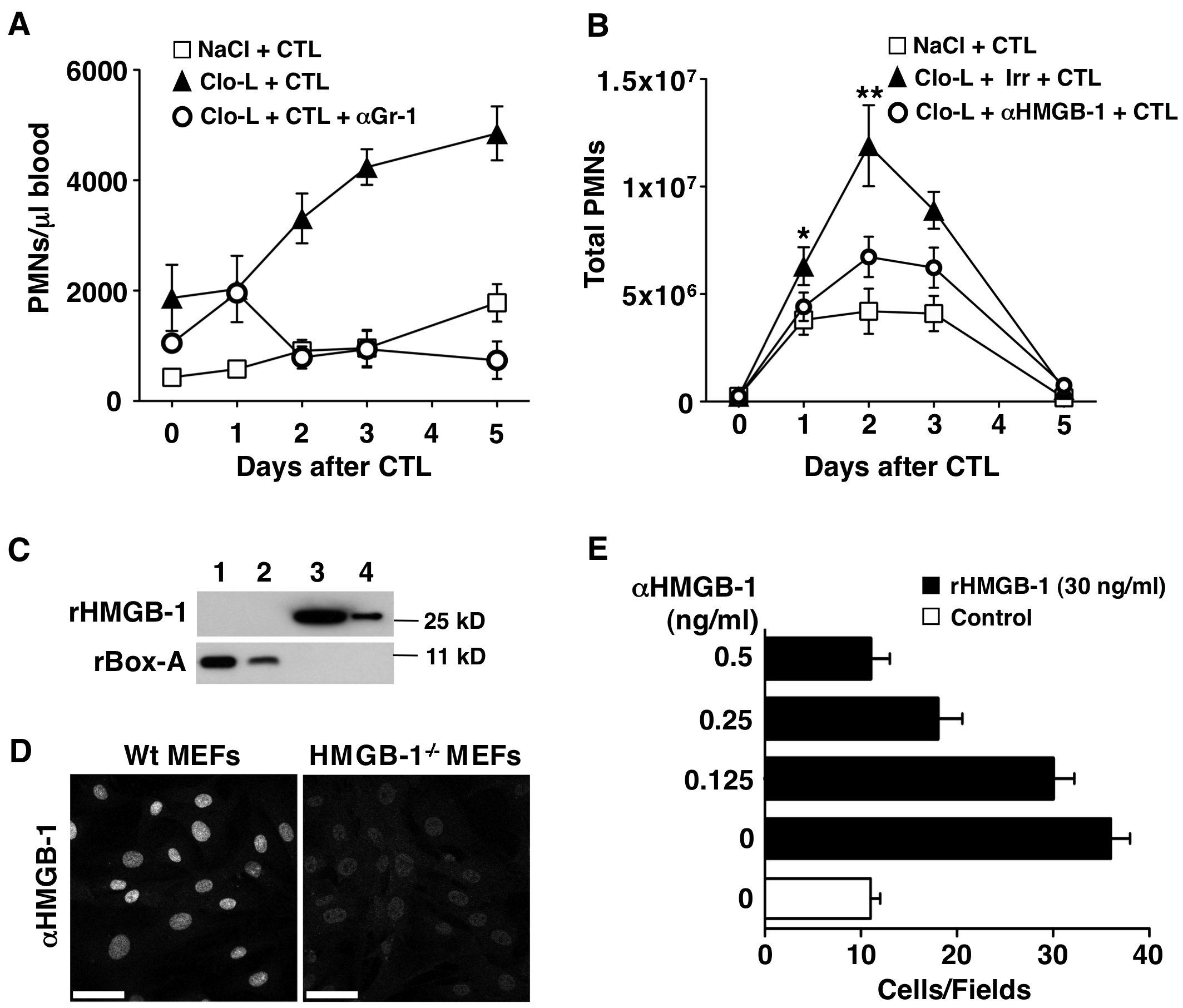

Supplement: Figure S6 — α-Gr-1 and α-HMGB-1 Abs reduce blood and liver PMNs counts. (A) Absolute number of Gr-1high CD11b+ PMNs in the blood of HBV replication-competent transgenic CTL-injected mice that received NaCl (white squares), Clo-L and irrelevant (Irr) Abs (black triangles) or Clo-L and anti-Gr-1 antibodies (αGr-1, black circles). n = 6. (B) Absolute number of Gr-1high CD11b+ neutrophils (PMNs) recovered from the livers of mice that received NaCl (white squares), Clo-L and irrelevant (Irr) Abs (black triangles) or Clo-L and the anti-HMGB-1 Ab DPH1.1 (αHMGB-1, black circles) along with the intravenous injection of 107 HBV-specific CTL. (C) Representative western blots of 500 ng (lane 1) or 100 ng (lane 2) of the recombinant Box-A fragment of HMGB-1 (negative control) and 500 ng (lane 3) or 100 ng (lane 4) of recombinant HMGB-1 incubated with either the α-HMGB-1 Ab DPH1.1. (top panel) or a control anti-Box-A fragment Ab control (bottom panel). (D) Representative micrographs of mouse embryonic fibroblasts (MEFs) derived from either wild type (wt) mice or HMGB-1−/− mice that were stained by immunofluorescence with the α-HMGB-1 Ab DPH1.1. Scale bar represents 20 µm. (E) Transmigration of 3T3 cells toward a HMGB-1 gradient in the presence of the indicated concentrations of the α-HMGB-1 Ab DPH1.1 was examined using a modified Boyden chamber assay. Each bar represents the mean number of migrated cells ± standard deviation of triplicate samples. (TIF) [file ppat.1002061.s006.tif]

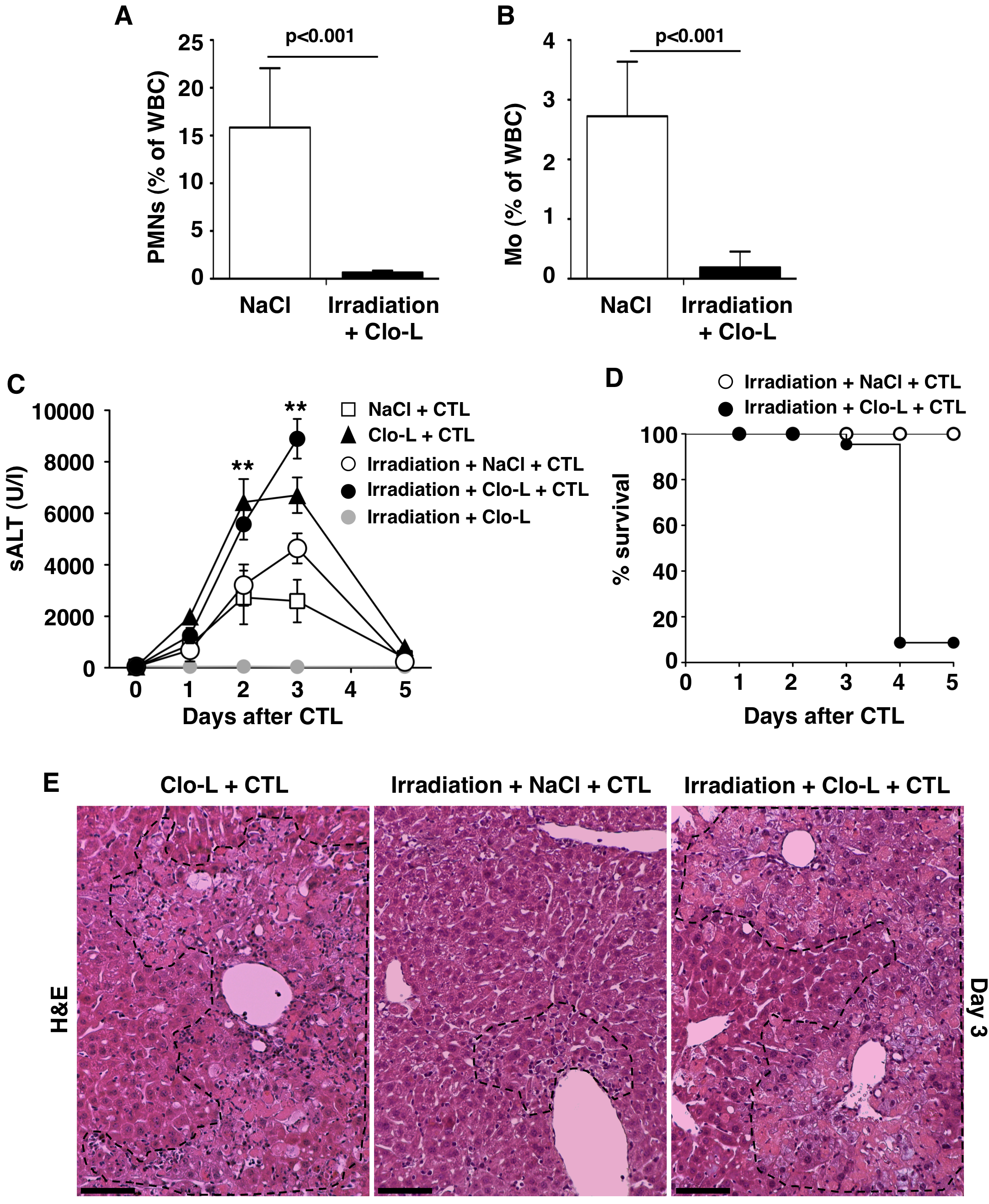

Supplement: Figure S7 — The effect of whole-body irradiation on liver disease severity. Clo-L treated and relative control HBV replication-competent transgenic mice were subjected to whole-body irradiation four hours prior to the transfer of 107 HBV-specific CTL. (A) Frequency of blood Gr-1high CD11b+ polymorphonuclear neutrophils (PMNs) and (B) Ly-6C+ monocytes recovered four hours after whole-body irradiation. n = 3. (C) Mean sALT activity (units/liter) measured at the indicated time points after CTL transfer in mice that received the indicated treatment. n = 6. (D) Kaplan-Meier survival curves of control (white) or Clo-L-treated (black) whole-body irradiated mice described in (B). n = 6. (E) Representative micrographs of hematoxylin/eosin-stained of Clo-L + CTL- (left), Irradiation + NaCl + CTL- (middle) or Irradiation + Clo-L + CTL- (right) treated HBV replication-competent transgenic livers, three days after intravenous injection of 107 HBV-specific CTL. Broken line delineates necroinflammatory foci. Note the abundant inflammatory infiltrate in the liver of mice treated with Clo-L + CTL (left), as opposed to what detected in the liver of mice treated with Irradiation + Clo-L + CTL (right). Note also the lack of large areas of damaged hepatocytes (and abundant inflammatory infiltrates) in irradiated mice not treated with Clo-L. All data are expressed as mean ± standard deviation and are representative of at least 2 independent experiments that gave similar results; differences between CTL-injected mice treated or not with Clo-L were not statistically significant unless otherwise indicated, ** p<0.001. (TIF) [file ppat.1002061.s007.tif]

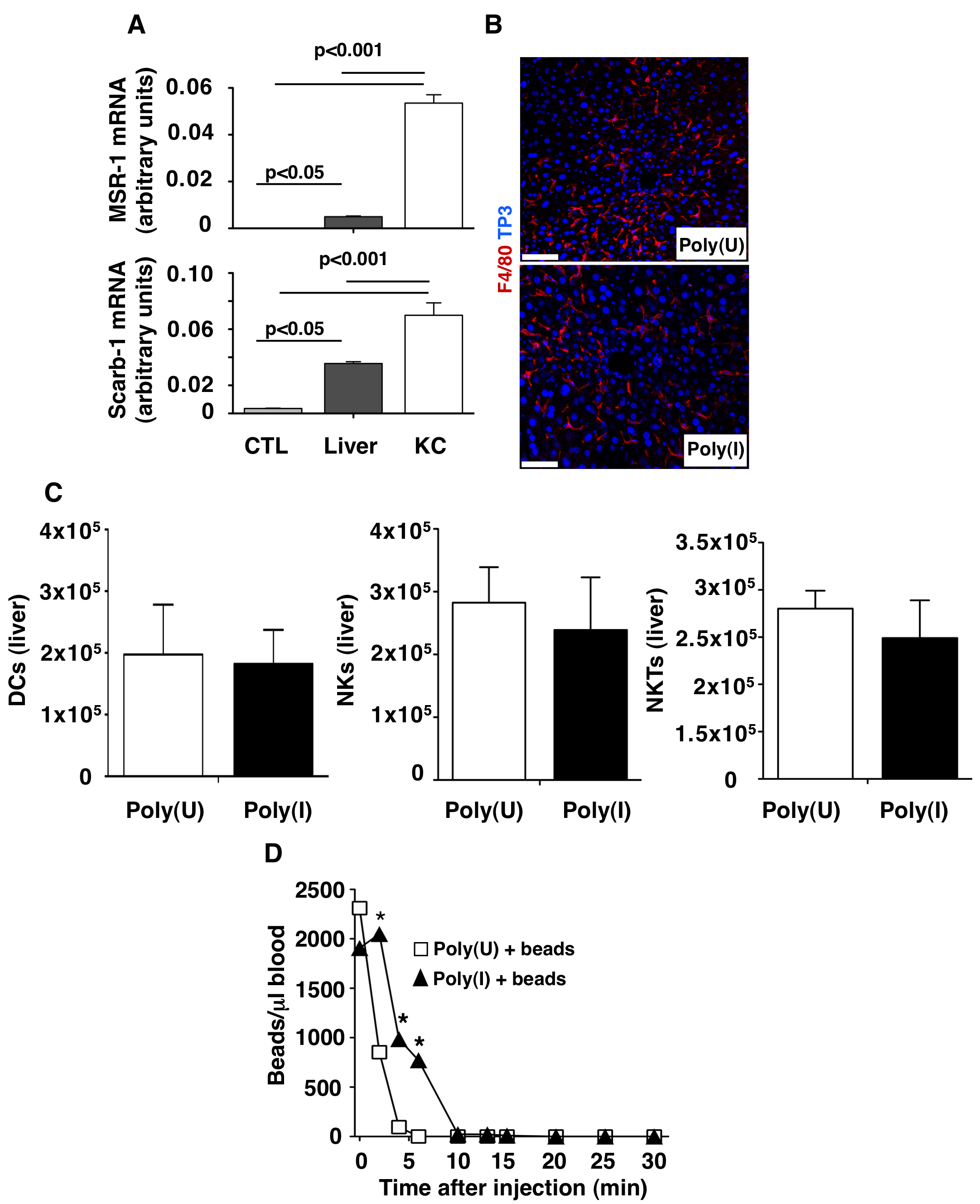

Supplement: Figure S8 — The scavenger receptor ligand Poly(I) inhibits liver phagocytosis. (A) Total RNA isolated from HBV-specific CTL, total liver RNA or KCs were analyzed by real time qPCR for the expression of macrophage scavenger receptor 1 (MSR-1) and scavenger receptor class b1 (Scarb-1). Results are expressed as arbitrary units after normalization for the housekeeping gene GAPDH. n = 6. (B) Representative confocal micrographs of HBV replication-competent transgenic livers 5 minutes after intravenous injection of Poly(U) (top panel) or Poly(I) (bottom panel). Anti-F4/80 staining in red, TO-PRO-3 (TP3) staining of nuclei in blue. Scale bar represents 150 µm. n = 3. (C) Absolute number of intrahepatic CD11chigh DCs, NK1.1+ CD3− NK cells and NK1.1+ CD3+ NKT cells recovered from the mice described in (B). n = 3. (D) Bead concentration in the blood of HBV replication-competent transgenic mice that received the indicated treatment. n = 6. All data are expressed as mean ± standard deviation and are representative of at least 3 independent experiments that gave similar results; differences between mice treated with Poly(U) or Poly(I) were not statistically significant unless otherwise indicated; * p<0.05. (TIF) [file ppat.1002061.s008.tif]
